# Altitude shapes gut microbiome composition accounting for diet, thyroid hormone levels, and host genetics in a subterranean blind mole rat

**DOI:** 10.3389/fmicb.2024.1476845

**Published:** 2024-11-01

**Authors:** Halil Mert Solak, Jakub Kreisinger, Dagmar Čížková, Efe Sezgin, Lucie Schmiedová, Marine Murtskhvaladze, Yoshiyuki Henning, Faruk Çolak, Ferhat Matur, Alexey Yanchukov

**Affiliations:** ^1^Department of Biology, Faculty of Science, Bülent Ecevit University, Zonguldak, Türkiye; ^2^Department of Zoology, Charles University, Prague, Czechia; ^3^Institute of Vertebrate Biology, Academy of Sciences of the Czech Republic, Brno, Czechia; ^4^Department of Food Engineering, Izmir Institute of Technology, Izmir, Türkiye; ^5^Institute of Ecology, Ilia State University, Tbilisi, Georgia; ^6^Institute of Physiology, University Hospital Essen, University of Duisburg-Essen, Essen, Germany; ^7^Department of Biology, Dokuz Eylül University, İzmir, Türkiye

**Keywords:** gut microbiome, diet, thyroid, altitude adaptation, high altitude, blind mole rats, 16S, 18S

## Abstract

The animal gut microbiome acts as a crucial link between the host and its environment, playing a vital role in digestion, metabolism, physiology, and fitness. Using 16S rRNA metabarcoding, we investigated the effect of altitude on the microbiome composition of Anatolian Blind Mole Rats (*Nannospalax xanthodon*) across six locations and three altitudinal groups. We also factored in the host diet, as well as host microsatellite genotypes and thyroid hormone levels. The altitude had a major effect on microbiome composition, with notable differences in the relative abundance of several bacterial taxa across elevations. Contrary to prior research, we found no significant difference in strictly anaerobic bacteria abundance among altitudinal groups, though facultatively anaerobic bacteria were more prevalent at higher altitudes. Microbiome alpha diversity peaked at mid-altitude, comprising elements from both low and high elevations. The beta diversity showed significant association with the altitude. Altitude had a significant effect on the diet composition but not on its alpha diversity. No distinct altitude-related genetic structure was evident among the host populations, and no correlation was revealed between the host genetic relatedness and microbiome composition nor between the host microbiome and the diet. Free thyroxine (FT4) levels increased almost linearly with the altitude but none of the bacterial ASVs were found to be specifically associated with hormone levels. Total thyroxine (TT4) levels correlated positively with microbiome diversity. Although we detected correlation between certain components of the thyroid hormone levels and the microbiome beta diversity, the pattern of their relationship remains inconclusive.

## 1 Introduction

The animal microbiome, which constitutes the microbial community within the gastrointestinal system, profoundly influences diverse aspects of the host, encompassing digestion, development, immunity, and energetics (Lindsay et al., 2020; McFall-Ngai et al., 2013; Suzuki, 2017). It also has the potential to enhance the host’s evolutionary capacity and fitness by shaping its phenotype (Alberdi et al., 2021; Henry et al., 2019; Suzuki, 2017; Henry et al., 2021). Humans, laboratory animals, and domestic mammals often serve as valuable models to study the microbiome’s importance in host ecology, evolution, health, and disease (Lin and Zhang, 2017; Lynch and Pedersen, 2016; Thursby and Juge, 2017; Wang et al., 2019; Bäckhed et al., 2007). While studying captive animals and humans is convenient, this approach has limitations, including the effect of controlled captivity conditions on the microbiome (Rogers et al., 2014; Roeselers et al., 2011; Van Leeuwen et al., 2020; Belheouane et al., 2020), lack or loss of heterogeneity in model (or held in captivity) hosts and their microbiome (Wang et al., 2014; Kohl et al., 2014), lack of environmental pressure (Chong-Neto et al., 2022), and variation in lifestyles and diets in humans (De Filippo et al., 2010) make it challenging to extrapolate the significance of findings to natural environments. Thus, employing natural models in research becomes not just a choice but a necessity whenever feasible to reveal the implication of host-microbiota interactions in ecological and evolutionary processes (Hird, 2017; Greyson-Gaito et al., 2020).

The microbiome is influenced by various factors, such as habitat variation (Amato et al., 2013), lifestyle (McKenzie et al., 2017), host diet (Pellizzon and Ricci, 2018; De Filippo et al., 2010; Kuang et al., 2022), host phylogeny (Ley et al., 2008), reproductive status of host (Nuriel-Ohayon et al., 2016), social interactions (Li et al., 2016c; Grieneisen et al., 2017) and environment (Rothschild et al., 2018; Chong-Neto et al., 2022; Ahn and Hayes, 2021). Among these factors, the environment holds significant importance as it can impact the physiology and immune responses of the host, while also playing a pivotal role in shaping dietary preferences and the selection process of the diverse pool of microbes in the surrounding environment. The microbiome can also help the host to adapt to new environments by modulating gene expression associated with nutrient metabolism, enhancing immune function, and providing essential nutrients (Bisschop et al., 2022; Petersen et al., 2023; Henry et al., 2021). In addition, microbiome diversity correlated positively with metabolic rate in a few studies, e.g., in wild and captive giant pandas (Zhu et al., 2011) and in hibernating brown bears (Sommer et al., 2016).

Highland environments are characterized by decreased atmospheric oxygen levels and colder temperatures, which poses various challenges to animals. Specifically, hypoxia tolerance and energy-efficient metabolism among species living at high altitude is the most common adaptation mechanisms (Storz and Moriyama, 2008; Storz et al., 2019; Cheviron et al., 2014; Schippers et al., 2012). Interestingly, the high-altitude adaptations do not seem costly, for example, humans living at high altitude have longer lifespans (Midha et al., 2023; Rogers et al., 2023). Recently, gut microbiome (hereafter GM) with specific diversity, composition, and function have been proposed as an additional factor that could contribute to high-altitude adaptation. For example, strong positive correlation has been demonstrated between the elevation and the proportion of anaerobic bacteria in the GM of wild house mice (*Mus musculus*) (Suzuki et al., 2018), which suggests that microbiota might contribute to the adaptation to hypoxia by helping to cope with low oxygen levels. Studies on wild pika (Ochotonidae) have demonstrated that an increase in altitude positively affects the diversity of gut microbial community, both at the host individual (alpha-diversity) and the population (beta-diversity) level (Li et al., 2019). Several species of wild ungulates on the Tibetan plateau have more diverse microbiomes, suggesting specific microbiome changes associated with high altitudes that facilitate extracting more energy from the plant diet (Ma et al., 2019).

Despite the growing body of research on the variation of microbiota with the altitude, it is not easy to disentangle which components of this variation represent genuine adaptations to high altitude environments. Apart from the environmental factors that systematically change with the altitude (e.g., oxygen level or temperature), there are multiple other modulators of gut microbiota that can co-vary with the altitude. One of them is host genetics, (Tabrett and Horton, 2020; Dąbrowska and Witkiewicz, 2016), which is a basis of evolutionary adapation (Suzuki et al., 2019). Because host species colonization history often follows the altitudinal gradient, the genome-wide population differentiation is expected to vary with altitude. The change in altitude also affects the composition of the ecological communities (Gale, 2004; Di Musciano et al., 2021; Lee et al., 2021), which in turn shape the diet of animals (De Filippo et al., 2010; Kuang et al., 2022). The diet stands as one of the primary factors affecting the microbiome composition. Finally, seasonal variaion might also affect the microbiome diversity and composition (Guo et al., 2021; Hu et al., 2018; Jiang et al., 2021; Fan et al., 2022).

Some study systems might be more suitable for disentangling the direct drivers of gut microbiota composition and diversity from those that simply co-vary with the altitude. Subterranean rodents, particularly *Nannospalax xanthodon* (Anatolian Blind Mole Rat, hereafter ABMR) possesses a unique combination of several traits that could offer an opportunity to disentangle such effects. The AMBR is an obligate subterranean rodent species (Arslan et al., 2016), setting it apart from many of its terrestrial and social rodent counterparts. This distinct ecological niche is likely to have profound implications for the composition and dynamics of its gut microbiota. Unlike social rodents that live in close-knit communities, ABMRs are solitary, each constructing an intricate network of underground tunnels and aggressively defending them from conspecifics, except for mating and raising young (Sözen, 2005; Nevo, 1979, 2007). In contrast to social rodents, this solitary lifestyle limits direct individual interactions, and thus slows down the exchange of microbial communities within the population. The subterranean environment, characterized by more stable temperature and humidity, can also be severely hypoxic when compared to the ambient atmospheric O2 levels. This effect could be exacerbated by the high altitude hypoxia, putting pressure on the aerobic microbes and favoring the anaerobic ones (Suzuki et al., 2018). Another potential factor relevant for gut bacteria is the increased concentration of carbon dioxide inside the AMBR tunnel networks, since some gut bacteria may compete for CO2 as a substrate (Nollet and Verstraete, 1996).

AMBR inhabits various regions of Turkey, from the warm Mediterranean coast of the Aegean Sea to the cold alpine climates of the Taurus Mountains and Eastern Anatolia. At the same time, its dispersing ability is expected to be quite limited compared to above-ground rodents. Slow dispersal, combined with small population size, is expected to allow ample time for the various types of environmental adaptation, which may include changes in microbiome composition, to manifest into notable differences between neighboring populations. Finally, the AMBR feeds on a wide range of plant species, predominantly those with nutrient-rich root parts, yet with a high fibre content (Sözen, 2005; Heth et al., 1989; Ülgen and Tavşanoğlu, 2024). This factor alone is expected to contribute to a high diversity of GM, offering a rich material for exploring the roles of the various factors listed above. To date, only three studies addressed the microbiome composition of the representatives of the *Nannospalax* genus (Sibai et al., 2020; Solak et al., 2023; Kuang et al., 2022), and just two of them were set in a natural environment (Kuang et al., 2022; Solak et al., 2023).

Notably, the ABMR is in fact a taxonomic complex of multiple, cytogenetically distinct, and potentially genetically isolated geographic populations (Arslan et al., 2016). At the same time, many (cyto) genetically uniform populations have continuous distributions occupying a range of diverse habitats. In this study, we focused on one the most striking example from the Central Taurus (Bolkar) mountains, where a single chromosomal race of ABMR (*“cilicicus”*) with a diploid chromosome number 2n = 58 occurs at the elevations from ca 1,000 m to 3,000 m above sea level (Sözen et al., 2006). Employing a culture-independent, advanced high-throughput metabarcode amplicon sequencing approach, we investigated: (i) the gut microbiota of ABMR using the 16S rRNA, (ii) its diet composition using 18S rRNA, (iii) thyroid hormone levels as an indirect proxy of the metabolic rate and (iv) the host genetic structure using microsatellite markers. The primary aim was to determine whether and how the altitude in conjunction with other factors influences the composition of the GM of ABMR.

## 2 Methods

### 2.1 Study setup and sampling

**Figure 1:**
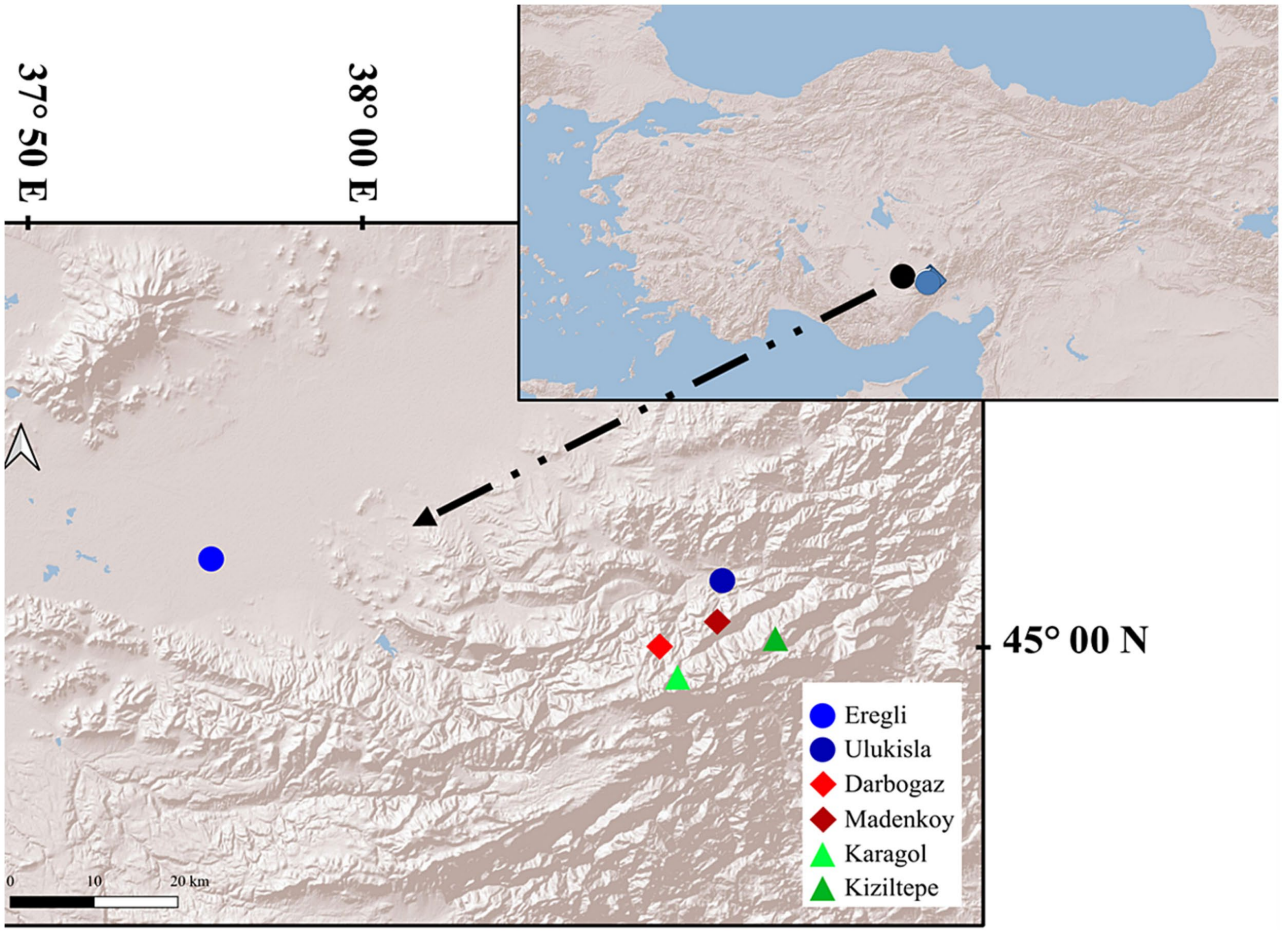
The sampling area in the Central Taurus mountains, Türkiye. The insert on the right shows the geographic location within the Anatolian peninsula.

Our sampling site is located in the Central Taurus Mountains in Türkiye, encompassing an altitudinal gradient ranging between 1,000 m asl and 3,000 m asl (Figure 1). The annual mean temperature and mean precipitation at the highest sampling location are 0.16°C and 744.6 mm, respectively, while they are 11°C and 344.7 mm at the lowest sampling location. Partial snow cover is present until June at high altitudes and may limit digging activities and access to water for these animals.

The sampling was conducted at three different altitude categories, each represented by two locations. The study area is about 460 km^2^, the distance between farther most localities (Eregli and Kiziltepe) is about 68 kilometers and the average distance between localities (except Eregli) is 8.5 kilometers. To minimize the influence of seasonal variation, the sampling was carried out during the same 2 months (late June to mid-July) in 2019 and 2021. From each locality, at least eight samples were collected, resulting in a total of 65 individuals. However, some samples failed during the PCR step of 16S and 18S rRNA library preparation. Sampling details are shown in Figure 1 and Table 1.

**Table 1:** Number of samples used in each analysis.

Altitude category	Locality	Elevation	Male	Female	Total	Microbiome	Diet	Thyroxine	Microsatellites
Low	Eregli	1,050 m	5	10	15	8	8	4	15
	Ulukisla	1,250 m	3	6	9	9	4	5	5
Middle	Madenkoy	1880 m	3	5	8	8	8	8	7
	Darbogaz	2000 m	3	5	8	7	4	0	8
High	Karagol	2,550 m	4	11	15	10	2	10	15
	Kiziltepe	2,900 m	1	11	12	9	10	0	12
		Total	19	48	67	51	38	28	63

Animals were live-captured by opening the burrow passageways at specific sections and blocking retreat when the animal mended the tunnel (Wertheim and Nevo, 1971). After recording body mass and sex, animals were euthanized and dissected. Approximately 1 ml of whole blood was taken from the heart, kept overnight at ~4\u00B0C, and centrifuged at 7 RPM to separate the serum. The serum samples and entire digestive tracts were placed on dry ice and kept frozen until transferred to a − 80°C freezer within several days. Additionally, a ~ 5 g caecum sample (both the content and the caecum wall) was stored in ethanol immediately upon dissection. The procedure was approved by the Animal Ethics Committee of Bülent Ecevit University (#91330202).

### 2.2 Genotyping of gut microbiome and diet content

A small (~5 g) piece from the terminal end of the caecum was collected and ~ 0.25 g of the tissue with its content was used for DNA extraction using the DNEasy PowerSoil Kit (Qiagen, Cat No: 47014) following the manufacturer’s protocol.

For the microbiome genotyping we used 17 individuals from low altitude, 15 individuals from middle altitude, and 19 individuals from high altitude, with a minimum of 7 samples per locality, on a total of 51 samples (Table 1). For the diet genotyping we used 12 individuals from low altitude, 14 individuals from middle altitude, and 12 individuals from high altitude, with a minimum of 2 samples per locality, on a total of 38 samples. On diet genotyping, 13 samples failed during the PCR amplification stage.

To amplify the V3-V4 variable region of the bacterial 16S rRNA gene, the universal primers S-D-Bact-0341-b-S-17(5’-CCTACGGGNGGCWGCAG-3′) and S-D-Bact-0785-a-A-21 (5’-GACTACHVGGGTATCTAATCC-3′) (Klindworth et al., 2013) were utilized. The 5′ ends of the primers were extended with inline barcodes to increase the multiplexing capacity (Supplementary Table S1). PCR reactions done in 10 μL volume, with 1x KAPA HIFI Hot Start Ready Mix (Kapa Biosystems, United States) and each primer at 0.2 μM and 4.6 μL of DNA template with the following cycling conditions: initial denaturation at 98°C for 5 min, followed by 30 cycles of 98°C (15 s), 55°C (20 s), and 72°C (40 s), and a final extension at 72°C for 5 min. The dual-indexed Nextera sequencing adapters were ligated during the second PCR, which is the same cycling conditions except it was performed in 20 μL volume, the concentration of each primer was 1 μM, 1.5 μL of the first PCR product diluted × 12.5 was used as a template and the number of PCR cycles was 12. The quantity and the expected length of the PCR products were evaluated by running them on a 1.5% agarose gel and successfully amplified bands were pooled equimolarly, and purified with SpriSelect beads (Beckman Coulter, United States). The resulting pool was subjected to size selection using the Pippin Prep automatic size selection system (Sage Science), targeting an amplicon size window of 520–750 bp. The pool of libraries was sequenced using MiSeq (Illumina, United States) and v3 chemistry (i.e., 2 × 300-bp paired-end reads) at the CEITEC Genomics Core Facility (Brno, Czech Republic).

To amplify 110–150 bp of eukaryotic 18S rRNA, a similar two-step PCR protocol was employed. We modified the primers from (Guardiola et al., 2015). F primer appended with 4–5 bp inline barcode to increase multiplexing possibilities and both primers appended with the “tail sequence” for priming the second PCR (Supplementary Table S2). Two elongation blockers were added to the first PCR to prevent amplification of (1) the mole rat DNA (Rodent_blckr: +G + T + C + C + C + C + C + A + A + C + T/3SpC3/; plus sign stands for LNA modification; 3SpC3 is C3 spacer at the 3′ end) and (2) the intestinal nematode common in rodents, *Syphacia* spp. (Syphacia_blckr: +T + G + T + C + T + G + A + A + A + T + A + C + T/SpcC3/). The final sequence of primers are: Guard_18S_F_longer: GATYTGTCTGGTTVATTCCGand Guard_18S_R_longer: CATCACAGACCTGTTATYGC. First PCR was performed in 10 ul reaction (1x KAPA HIFI Hot Start Ready Mix, 0.3 μM of each primer, 1uM of Rodent_blckr, 2 uM of Syphacia_blckr and 1.4 ul of caecal DNA), with 3 min at 95°C and 34 cycles of 98°C (20 s), 56.5°C (15 s) and 70°C (15 s), and a final extension at 70°C (30 s). The second PCR was performed in 15 uL (1x KAPA HIFI Hot Start Ready Mix, 0.5 uM of each indexed primer, and 1 uL of the first-PCR product as template), with 3 min at 95°C and 12 cycles of 98°C (20 s), 55°C (15 s) and 72°C (15 s), and a final extension at 72°C (3 min). Each PCR was performed in a technical duplicate. PCRs were quantified using 2% agarose gel electrophoresis and pooled equimolarly. Fragments between 240 and 360 bp were extracted using PippinPrep and sequenced with Illumina Nextseq, 150 bpPE reads at the CEITEC Genomics Core Facility (Brno, Czech Republic).

To account for possible amplification stochasticity, each sample was amplified and genotyped twice. In the subsequent analyses, the data from the duplicates were treated as individual samples.

### 2.3 Individual genotyping of the host

For the host genotyping, we used 23 individuals from low altitude, 15 individuals from middle altitude, and 27 individuals from high altitude, with a minimum of 5 samples per locality, on a total of 65 samples (Table 1).

The genotyping protocol utilized a combination of seven microsatellite markers sourced from (Popa et al., 2014) and six additional microsatellite markers from (Karanth et al., 2004). Consequently, a total of 13 variable microsatellite markers were employed for the genotyping analysis (see Supplementary Table S3). Multiplex PCR was performed with FAM and HEX fluorescent-labeled primers, using a QIAGEN Multiplex PCR Kit (QIAGEN, Cat. No: 206143). This enabled the amplification of multiple targets within a single reaction. Fragments were differentiated by the respective fluorescent labels as well as by their expected sizes.

PCR reactions were performed in PCR plates in 7 μL reaction volumes (3 μL of Multiplex PCR Buffer, 0.6 μL of Q-solution, 1.5 μl of DNA, 0.5 to 1 μl of primer, and distilled water). Notably, the HEX-labeled primers required higher concentrations (10 picomoles) compared to FAM markers (5 picomoles) for optimal performance.

The PCR temperature profile consisted of an initial denaturation step at 95°C for 15 min, followed by 10 cycles of denaturation at 93°C for 40 s, annealing at 60°C for 40 s (with a decrease of 0.4°C per cycle), and extension at 72°C for 80 s. This was followed by 20 cycles of denaturation at 93°C for 30 s, annealing at 56°C for 40 s, extension at 72°C for 80 s, and concluded with a final extension step at 60°C for 30 min.

For fragment analysis, we employed the GeneScan™ 500 LIZ™ dye Size Standard (Applied Biosystems™, Cat. No: 4322682). Initially, the size standard was diluted with Hi-Di™ Formamide (Cat. no. 4311320) at a ratio of 40 size standard to 1,000 formamide. Subsequently, 10 μL of the diluted standard was added to each well, followed by the addition of 2–3 μL of PCR product. The plate was subjected to a thermal cycler at 95°C for 3 min, followed by immediate chilling. Fragment analysis was performed using the 3130XL Genetic Analyzer (Applied Biosystems™), with allele identification carried out using Gene Mapper Software (version: 5.0).

### 2.4 Thyroxine levels quantification assays

Using the serum from four localities among 3 altitude groups (Table 1), we measured free fractions of thyroxine and triiodothyronine (fT4 and fT3) as well as total thyroxine and triiodothyronine (TT4 and TT3) using commercial ELISA kits according to manufacturer’s instructions (DRG Diagnostics, Marburg, Germany: FT4 - EIA 2386; FT3 - EIA 2385; TT4 - EIA 4568; TT3 - EIA 4569).

### 2.5 Bioinformatic analyses

Following demultiplexing and trimming of the raw 16 s rRNA and 18 s rRNA sequencing data using Skewer (Jiang et al., 2014) reads with low quality were eliminated by setting the expected error rate per paired-end read >1 (Jiang et al., 2014).

The quality-filtered reads were denoised with DADA2 software (Callahan et al., 2016), resulting in an abundance matrix containing the number of reads for each amplicon sequence variant (ASV) in each sample. The UCHIME software (Edgar et al., 2011) was employed to identify and remove sequence chimeras, with gold.fna database^1^ serving as a reference for chimera filtering. For 16S rRNA bacterial ASV annotation in the DADA2 software, the Silva database version 138.1 (updated in March 2021; Quast et al., 2013) was used as a reference. For the 18S rRNA dataset, for each ASV, the top 200 Blastn hits were downloaded from the NCBI nucleotide database (Camacho et al., 2023) and used to construct the custom reference database. The ASV taxonomy was then assigned using the RDP classifier as previously described (Quast et al., 2013). Finally, the *phyloseq* (McMurdie and Holmes, 2013) package was utilized to construct a comprehensive database containing the Amplicon Sequence Variants (ASVs) table, ASV sequences, taxonomic annotations at phylum and family and genus level (when possible), and phylogeny for both datasets.

### 2.6 Microbiome and diet

The microbiome database comprised 1,103,094 high-quality sequences grouped in 4841 non-chimeric ASVs. The number of sequences in the microbiome database per sample varied between 8,936 and 30,237. The diet database comprised 2,392,247 high-quality sequences grouped in 140 non-chimeric ASVs. The number of sequences in the diet database per sample varied between 515 and 205,099. For the rarefaction of the ASV table, “phyloseq_mult_raref_avg” function from the *metagMisc* package was used with 100 iterations, which provides robustness by applying repeated subsampling. Using the minimal sequencing depth as the rarefaction threshold, we ensured even sequencing depth per sample and utilized the down-sampled dataset for further analysis unless otherwise stated. All the statistical analyses were done in R version 4.2.2.^2^

The procrustes test (Procrustes Rotation of Two Configurations in *vegan* pack (Oksanen et al., 2024)) was used to compare duplicates and for both datasets procrustes test showed the composition was consistent across all duplicates (number of permutations = 999, procrustes sum of squares (m12 squared) = 0.00414, Correlation in a symmetric Procrustes rotation = 0.9979, *p*-value = 0.001).

We employed the exact observed number of ASVs, Shannon, and Simpson indices to estimate alpha diversity using the “estimate_richness” function in the *phyloseq* package (McMurdie and Holmes, 2013). To compare alpha diversities between altitudinal groups, we used the “wilcox.test” function (hereafter WT, *stats* package) (Bauer, 1972). Per-sample Shannon and Simpson diversity indices were used as response variables in the Generalized Linear Mixed Models with Gaussian distribution (hereafter GLMM, *glmmTMB* package; Brooks et al., 2017), with the altitude category (low, middle, and high) included as a fixed variable and sampling location included as a random variable.

As a measure of beta diversity, we employed the Bray–Curtis dissimilarity index, which focuses on relative abundances using the “distance” function with a specified “bray” method (*phyloseq* package). We visualized the between-sample divergence pattern using Principal Coordinate Analysis (PCoA). Furthermore, we applied PERMANOVA (Permutational Multivariate Analysis of Variance Using Distance Matrices, “adonis2” function from the *vegan* package) to test for differences in the gut microbiota composition between altitudes. Additionally, to take account of the possible effect of the sampling localities, we used the first two principal components of the Bray-Curtis PCoA as response variables in the GLMM analysis, with altitude category (low, middle, and high) included as a fixed variable and sampling location included as a random variable. Furthermore, the effect of fixed variables was tested using likelihood ratio tests (“anova” function, *stats* package). Finally, we employed the MDMR (Multivariate Distance Matrix Regression, *mdmr* package (McArtor et al., 2017)) with the Bray-Curtis distance matrix as a response variable, altitude category as a fixed variable, and sampling location as a random variable. Then, for both alpha and beta diversity measures, we tested the effects of sex, sampling year, and body mass using the same statistical approach. We checked for the possible correlation between microbiome and diet composition. First, the samples not present in both datasets were excluded. Then the Mantel Test (*ape* package; (Mantel, 1967; Paradis and Schliep, 2019)) was employed to check the correlation between diet and microbiome composition.

We used the “DA.kru” function in the *DAtest* package for calculating Kruskal-Wallis analysis (Russel88/DAtest on GitHub (Russel et al., 2018)) to compare the differential abundances of bacterial phyla and families among different altitudes. The *p*-values from “DA.kru” function were adjusted by “FDR” method (Benjamini and Hochberg, 1995) as default. To explore the relative abundances of ASVs that significantly differ among altitudinal groups or sampling localities, we used the *DESeq2* package (Anders and Huber, 2010). Additionally, we specifically focused on ASVs that exhibit higher abundance in one location or a select few, while being nearly or entirely absent in others. To do that, we employed “estimateSizeFactors” function in the *DESeq2* package with *LTR* test parameter (Likelihood ratio test (chi-square test) for GLMs).

Finally, we predicted high-level bacterial characteristics using BugBase4 (Ward et al., 2017) based on 16S rRNA data, following the online instructions. These phenotypes included Gram Positive, Gram Negative, Biofilm Forming, Pathogenic Potential, Mobile Element Containing, Oxygen Utilizing, Oxidative Stress Tolerant, and Facultatively Anaerobic bacteria. The relative frequencies of predicted phenotypes were then tested for altitudinal difference using Kruskal-Wallis Test (“kruskal.test” in *stats* package (Hollander et al., 2013)) and the p-values were corrected using “p.adjust” function in *stats* package with the FDR method.

### 2.7 Host genetics

The individual genotypes were handled in *adegenet* package (Jombart, 2008) as a “genind” object. We calculated Nei’s pairwise Fst (Nei, 1973) between all pairs of populations by “pairwise.neifst” function *hierfstat* package, (Goudet, 2005). Then, we calculated the mean proportions of shared alleles by using the “propShared” function (*adegenet* package), averaged per respective sampling localities. Then, allelic richness for each altitudinal group was calculated using “allelic.richness” function (*hierfstat* package) and compared with “t-test” function (*stats* package). Finally, we run AMOVA (Analysis of Molecular Variance) for altitudinal groups and sampling localities “using poppr.amova” function in *poppr* package (Kamvar et al., 2014), and generated the p-values using “randtest” function in *ade4* package.

STRUCTURE software (V2.3.4, (Pritchard et al., 2000)) was used to reveal the population genetic structure using the Admixture Model with 5,000 burnin, 50,000 MCMC replicates after burnin, 5 iterations, and correlated allele frequencies. The analyses run with and without sampling locality as prior, and a number of inferred genetic clusters (K) is set to a range between 1 to 6. The resulting output is reviewed on the Structure Harvester website (v.0.6.94, (Earl and vonHoldt, 2012)).

Using the “coancestry” function (*related* package; Pew et al., 2015), we calculated the empirical relatedness between same-population individuals according to Li et al. (1993) to seek for possible correlation between kinship and microbiome composition. Only those samples present in both datasets were included. The pairwise matrix of genetic distances (measured as 1-relatedness) was computed using the “mat_gen_dist” function, utilizing the “PCA” method (*graph4lg* package; Savary et al., 2021). Subsequently, Euclidean distance matrices were generated for each population. The Bray–Curtis dissimilarity distance matrices were constructed on the individual microbiome data, and the Mantel Test (*ape* package; Mantel, 1967; Paradis and Schliep, 2019) was employed to check for the correlation between host kinship and microbiome composition. Additionally, “grouprel” function (*related* package) is employed to calculate average within-group relatedness to compare with expected relatedness in simulated populations with 100 iterations (ie. 100 parent-offspring, 100 full siblings, 100 half siblings, and 100 unrelated).

### 2.8 Thyroid hormone levels

Concentrations of fT4, TT4, fT3, and TT3 were analyzed with multiple linear regression models with altitude, sex, and weight as explanatory variables. All data were tested for normal distribution using three normality tests (Anderson-Darling, D’Agostino-Pearson omnibus, and Shapiro Wilk). Since fT4 concentrations were not normally distributed, fT4 concentrations were log-transformed before analyses. We first calculated one model with main effects (model 1) and one model with two-way interactions (model 2) to identify the best-fitting model. The Akaike information criterion (AIC) was used to estimate model fit. Based on the best-fitting model, explanatory variables with significant effects on hormone concentrations were identified. These analyses were conducted using GraphPad Prism (vers. 9.3.1, San Diego, CA, United States).

Effect of altitude on hormone levels was tested using ANOVA. Since this approach did not take into account the random effect of sampling altitude, we used GLMMs and MDMRs with alpha diversity metrics as response variables, hormone measurements as fixed, and sampling locality as a random variable. For the beta diversity, we employed GLMMs and MDMRs using two principal components of the Bray-Curtis PCoA as response variables, and hormone levels as fixed variables, and the sampling locality and altitude as a random effect. Finally, we use the *DA.test* package to see if there is correlation between hormone levels and differential abundance of a bacterial taxa.

## 3 Results

### 3.1 Gut microbiome

We successfully genotyped the 16S rRNA amplicons of a total of 52 ABMR caecum samples in duplicates. For subsequent analyses, the data from duplicates were merged based on the consistent coverage of ASVs between duplicates, meaning that only the ASVs found in both duplicates were retained.

**Figure 2:**
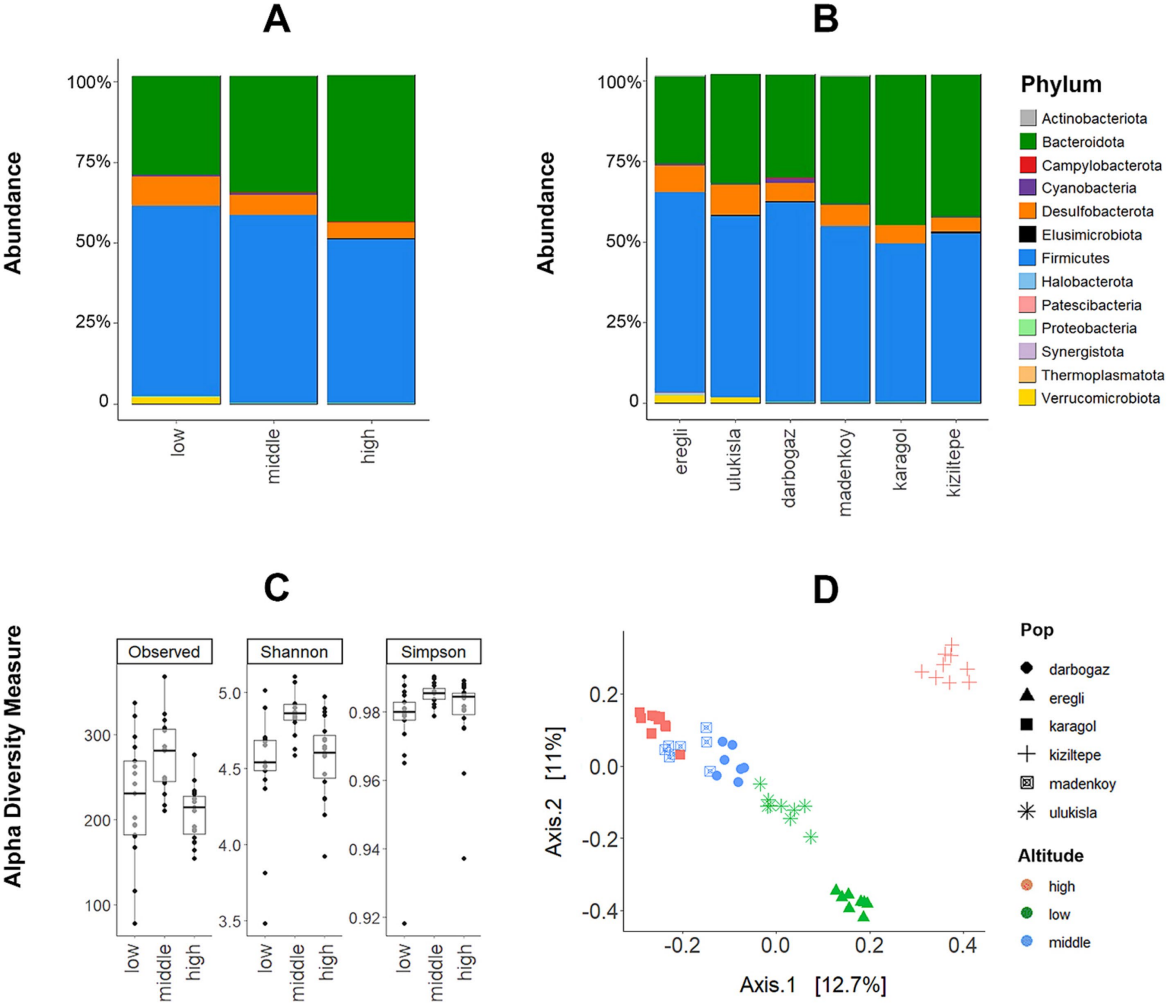
(A) The relative abundance of dominant bacterial phyla among the sampling localities and (B) altitudinal groups (X-axis represents % of the abundance of all reads). (C) Variation in the GM diversity (number of observed ASVs, Shannon, and Simpson diversity indices) across altitudes (*p*-values are given from WT analysis). (D) PCoA plot on Bray–Curtis dissimilarity metric showing the divergence between gut microbiota from altitudinal groups.

The microbiome database was dominated by Firmicutes (53% of all reads), Bacteroidota (36%), and Desulfobacterota (0,7%). Another 9 phyla detected were: Patescibacteria, Cyanobacteria, Elusimicrobiota, Campylobacterota, Proteobacteria, Actinobacteriota, Synergistota, Halobacterota, and Thermoplasmatota had low abundances (<1% reads, Supplementary Figure S1). At the family level, the data was dominated by Muribaculaceae (35%), Lacnospiraceae (28%), Christensenellaceae (~5%), Ruminococcaceae (~5%), Desulfovibrionaceae (~5%), Oscillospiraceae (~5%) and 29 other bacterial families with less than 1% abundance (Supplementary Figure S1). The relative abundances of bacterial phyla were visualized for each sample, sampling location, and altitudinal group (Figures 2A,B) and individual microbiome variability shown in Supplementary Figure S2.

### 3.2 The Alpha and Beta diversity of microbiome

The alpha diversity of the gut microbiota in ABMR exhibited variations across different sampling locations and altitudinal groups. Specifically, the animals inhabiting the middle altitude demonstrated a more diverse composition of the microbiome compared to those at low and high altitudes (Figure 2C).

In the case of all three alpha diversity metrics (number of observed ASVs, Shannon and Simpson diversity indices), the WT analysis revealed that samples from the middle altitude group exhibited significantly higher levels of among-sample diversity compared to the other altitudinal groups. Moreover, the WT analysis indicated a significant difference between the middle and high altitudes, except for the Simpson diversity index (*p*-values for each test are shown in Figure 2C). GLMM analyses revealed that the likelihood ratio test comparing the two models was not significant for the Shannon index (ΔD.*F* = 2, *χ*^2^ = 4.6772, p-value = 0.09), and Simpson index (ΔD.F = 2, *χ*^2^ = 2.4964, p-value = 0.28), but significant for the number of observed ASVs (ΔD.F = 2, *χ*^2^ = 10.571, p-value = 0.005). While WT analyses revealed significantly higher bacterial diversity at middle altitudes using all three alpha diversity metrics, GLMM showed significance only for the number of observed ASVs. It is important to note that, unlike GLMM, WT analyses do not account for the effect of sampling locality.

The PERMANOVA results demonstrated significant differences between the altitudinal groups in terms of Bray-Curtis dissimilarities (*F*-value = 4.6839, df = 2, *p*-value = 0.001, and *R*^2^ = 0.16). Furthermore, we employed the first two axes of the Bray-Curtis-based PCoA as response variables in a GLMM analysis to assess the altitude’s effect. The first PCoA axis mostly corresponded to the elevation gradient, separating the three altitude categories. A notable exception to this trend was the high-altitude site of Kiziltepe, as indicated by the data points in the top right corner of Figure 2D, which clustered far apart from all other data points. At the same time, the second PCoA axis represented the elevation gradient more clearly, with data points at the bottom of Figure 2D corresponding to low-altitude samples and those at the top corresponding to high-altitude samples. In the GLMM analyses, the likelihood ratio test comparing the two models for the first PCoA axis was not significant (*χ*^2^ = 1.6172, df = 2, *p* = 0.44) which is most probably due to the Kiziltepe samples which clustered far from the rest, especially along the first axis. However, the comparison for the second PCoA axis indicated that the categorical model significantly enhanced the explanation of variation in the ABMR gut microbiota (*χ*^2^ = 9.9051, df = 2, *p* = 0.007). Finally, the results from MDMR analyses also showed a significant effect of altitude (df = 2, *p*-value =0.0006). All of the above patterns remained significant when the data collected in each year were analysed separately, but at the same time, the sampling year was itself a significant factor in models considering altitude and sampling localities as random variables (GLMM: *χ*^2^ = 10.423, df = 1, *p*-value = 0.001; MDMR: df = 1, *p*-value =0.00001). Neither GLMM for sex and Shannon diversity considering sampling locality and altitude as random variables (*χ*^2^ = 1.6125, df = 1, *p*-value = 0.20) nor GLMM for sex and beta diversity (i.e., the first PCoA axis; *χ*^2^ = 0.0132, df = 1, *p*-value = 0.90) were significant. Similarly, GLMM for host body mass and Shannon diversity considering altitude and sampling localities as random variables (*Z*-value = 0.542, SE = 0.00091, *p*-value = 0.55) and GLMM for host body mass and beta diversity (i.e., the first PCoA axis; *χ*^2^ = 1.581, df = 1, *p*-value = 0.21) found to be insignificant.

### 3.3 Differential ASV abundance testing

Anayses within the *DA.test* package revealed a significant effect of the altitude on the relative abundance of bacterial phyla and families. Specifically, the relative abundance of Bacteroidota increased with altitude, whereas Firmicutes and Desulfobacterota were significantly more abundant at low altitudes, and Verrucomicrobiota was only found at low altitudes (Table 2; Figure 2A).

**Table 2:** Comparison of differential abundances of bacterial phyla between altitudes.

Phylum	Kruskal-Wallis *p* value	Adjusted *p* value (fdr)	Relative abundance among altitudes
Bacteroidota	0.00003	0.0004	**high > middle > low**
Firmicutes	0.007	0.03	**low > middle > high**
Desulfobacterota	0.01	0.04	low>high>middle
Verrucomicrobiota	0.0003	0.002	Only found at low

The last column indicates relative abundance among altitudinal groups (e.g., high > middle > low represents higher relative abundance at high altitude compared to middle altitude, and higher at middle altitude compared to low altitude). The gradual increase or decrease between relative abundance with altitude is marked as bold.

At the bacterial family level, we observed that the relative abundances of Muribaculaceae, and Unclassified Bacteroidales, increased with altitude, while the relative abundances of Lacnospiraceae, and Ruminococcaceae decreased. Additionally, Christensenellaceae was significantly more abundant at high altitude but less abundant at middle altitude, and Akkermansiaceae was only found at low altitude in both localities (Table 3).

**Table 3:** Comparison of differential abundances of bacterial families between the altitudes.

Family	Kruskal-Wallis *p* value	Adjusted *p* value (fdr)	Relative abundance among altitudes
Muribaculaceae	0.00005	0.002	**high > middle > low**
Lachnospiraceae	0.0001	0.003	**low > middle > high**
Akkermansiaceae	0.0003	0.005	Only found at low
Christensenellaceae	0.0006	0.007	low>high>middle
Unclassified Bacteroidales	0.002	0.02	**high > middle > low**
Ruminococcaceae	0.003	0.02	**low > middle > high**
Lactobacillaceae	0.004	0.03	high>low>middle
Butyricicoccaceae	0.005	0.03	middle>high>low

The last column indicates relative abundance among the altitudinal groups (e.g., high > middle > low). The statistically significant results are marked in bold.

The DESeq2 analysis revealed a significant effect of the sampling locality on the relative abundances of 51 ASVs (Table 4; Supplementary Figure S3). Twenty four of them were overabundant in one or in a few localities but significantly underrepresented in all the other localities (Table 4). Among these, Kiziltepe exhibited the highest number of ASVs with significant differential abundance (n = 12), while both high-altitude localities collectively harbored 19 significant ASVs. Intriguingly, no such ASVs were detected in the middle altitude localities. These DESeq2 results correspond to alpha diversity analyses, which also suggest that the middle altitude samples comprise a blend of two other altitudinal groups.

**Table 4:** Number of ASVs with significant abundance difference across sampling localities.

Altitude	Locality	No. of ASVs
Low	Eregli	3
	Ulukisla	2
Middle	Madenkoy	0
	Darbogaz	0
High	Karagol	4
	Kiziltepe	15

### 3.4 Bacterial characteristics prediction

**Figure 3:**
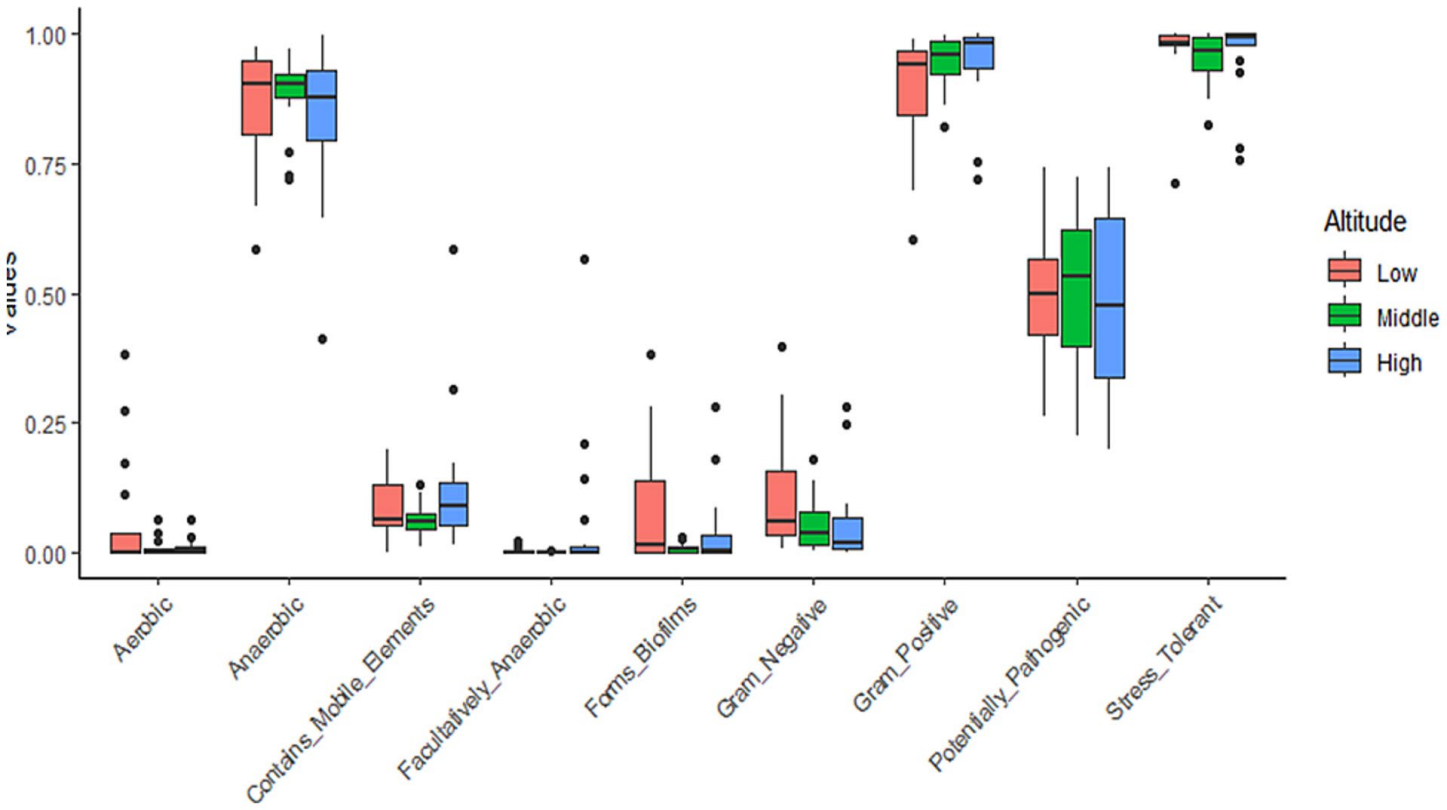
Variation of predicted bacterial phenotypes among the altitudinal groups. Values in the y-axis represent the BugBase bacterial phenotype prediction.

We used BugBase, a tool for measuring high-level phenotypes in the microbiome, to assess changes in bacterial phenotypes across different altitudinal groups (Figure 3). The ‘Facultatively Anaerobic’ phenotype was the only one that displayed a significant difference (Kruskal-Wallis fdr adjusted *p*-value = 0.049), attributed to its prevalence at the high altitude. Note that almost all ASVs that contributed to this phenotype belonged to the bacterial phylum Firmicutes, which itself was more abundant at the high altitude (Figure 2A).

### 3.5 Diet composition

We successfully genotyped the 18S rRNA amplicons from 38 ABMR caecum samples (12 low, 14 middle, and 12 high altitude) in duplicates. Similar to the 16S results, the data from duplicates were merged based on the same coverage of ASVs between duplicates, and the data from duplicates were treated as individual samples.

The raw dataset was clustered in four phyla: Arthropoda, Basidiomycota, Chordata, and Streptophyta. However, considering the ABMRs are herbivorous rodents (Sözen, 2005), the database was filtered by excluding the host genome and other possible contamination-caused sequences from non-plant phyla, retaining only those belonging to plants (Streptophyta). This resulted in approximately two-thirds of the reads being retained for further analysis. At the order level, the data was dominated by Asterales (42.7%) and Brassicales (21%), Apiales (15.21%), Fabales (10.37%), Asparagales (7.92%), Rosales (6.61%), and as well as 12 other plant orders with less than 5% relative abundance. Due to the relatively short sequence lengths of our 18S rRNA libraries which become increasingly unreliable when assigning low-level taxa and lack of complete reference database focused on plant 18S rRNA, we only resolved the diet taxonomy down to the order level.

**Figure 4:**
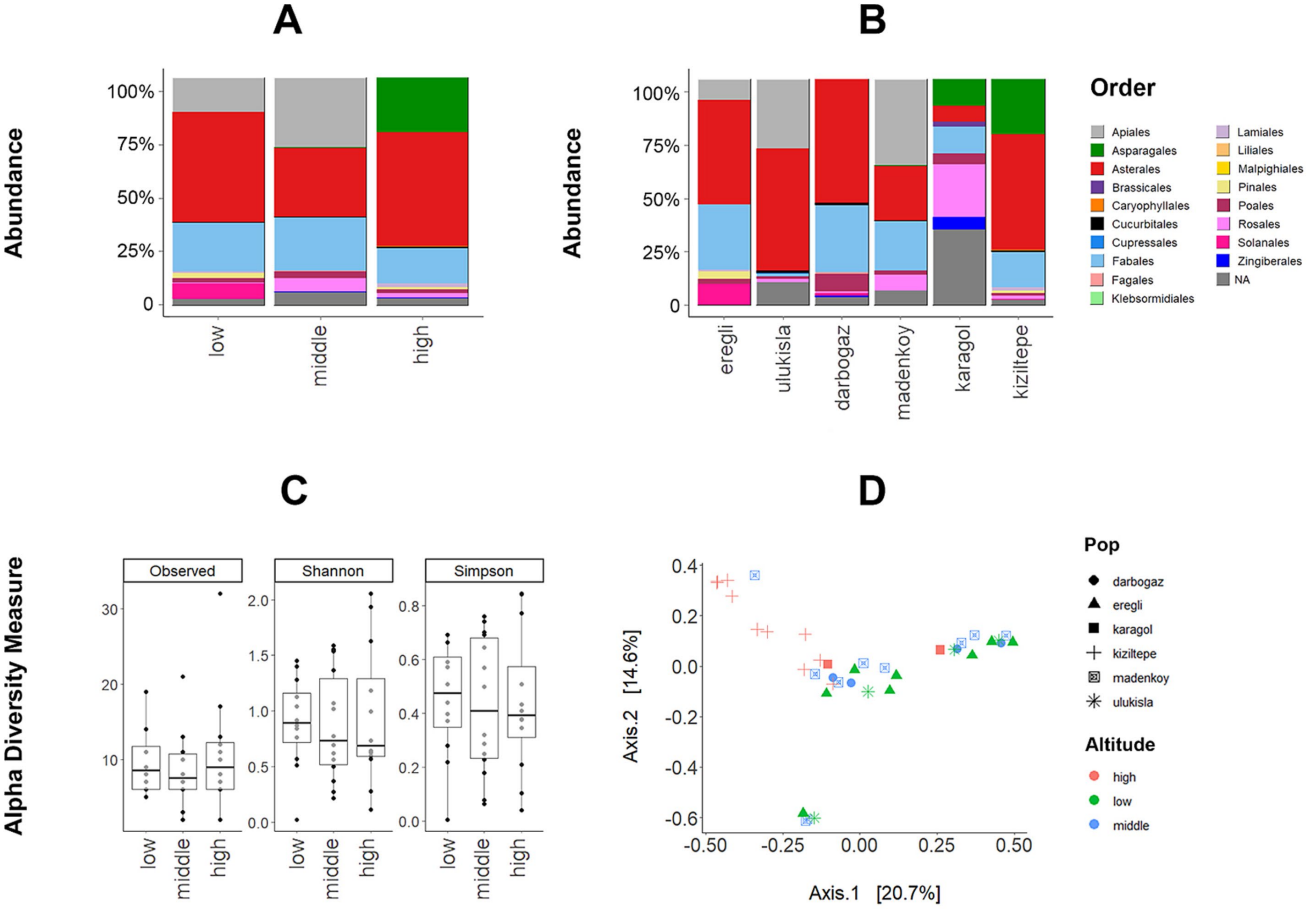
(A) The relative abundance of dominant plant orders among the sampling localities and (B) altitudinal groups (X-axis represents % of the abundance of all reads). (C) Box-plot showing variation in diet diversity (number of observed ASVs, Shannon, and Simpson diversity indices) between altitudes. Both WT and GLMM showed no significant difference between altitudes. (D) PCoA of Bray–Curtis dissimilarity in diet composition among the individuals, altitudes, and localities.

The relative abundances of plant orders across altitudinal groups and sampling locations are illustrated in Figures 4A,B. Asterales and Fabales were prevalent across all groups, whereas Apiales were exclusively observed at low and middle altitudes, with significantly higher abundance at the middle altitude (Kruskal-Wallis fdr adjusted *p*-value = 0.05). In contrast, Asparagales were observed exclusively at the high altitude, consistent with the *DA.test* results (Kruskal-Wallis fdr adjusted *p*-value <0.0001).

### 3.6 The Alpha and Beta diversity of diet

The observed number of ASVs, Shannon, and Simpson diversity measures did not significantly differ by altitude (Figure 4C). This result was supported by both WT and GLMMs (both *p*-values >0.05). Nevertheless, all three indices displayed the widest observable range at the middle altitude (Figure 4). After examining the variation in Bray–Curtis dissimilarity index of the diet composition with PCoA, PERMANOVA (*p*-value = 0.001, *R*^2^ = 0.14, and *F*-value = 2.8517), the GLMM for the first PCoA axis (*χ*^2^ = 7.6772, df = 2, *p* = 0.021), and MDMR for PCoA axes (df = 2, *p*-value = 0.00001) showed a significant effect of the altitude (Figure 4D). In the PCoA, the high-altitude animals clustered separately, while the low and the middle altitudes overlapped to some extent. There was no significant effect of the sex, body mass, and sampling year on the diet of ABMR. The Mantel Test indicated no significant correlation between the diet and the microbiome composition (*p*-value = 0.20, *r* = 0.03).

### 3.7 Host genetics

A total of 65 samples were genotyped at the six loci reported in (Karanth et al., 2004), with 6.81% missing data, but only the samples collected in 2019 were genotyped for the seven loci reported in (Popa et al., 2014), with 25% missing data. The mean allelic richness values calculated in low, middle, and high altitude groups were 1.636348, 1.735554, and 1.603827, respectively. There was no significant difference in mean allelic richness among the altitudinal groups (t-test p-values >0.05). The mean allelic richness at the sampling locality level ranged between 1.352573 (Kiziltepe) and 1.721504 (Madenkoy), and the only significant difference was observed between Eregli and Kiziltepe (t-test p-value = 0.01). The pairwise Fst and proportion of shared alleles between populations are shown in Table 5.

**Table 5:** Genetic differentiation among the sampling localities.

X	Eregli (L)	Uluskisla (L)	Darbogaz (M)	Madenkoy (M)	Karagol (M)	Kiziltepe (M)
Eregli	0	0.39	0.48	0.41	0.40	0.38
Ulukisla	0.059 (−0.011–0.187)	0	0.39	0.41	0.39	0.44
Darbogaz	0.056 (**0.014**–0.095)	0.116 (−0.005–0.220)	0	0.52	0.51	0.45
Madenkoy	0.028 (−0.013–0.084)	−0.025 (−0.070–0.029)	0.065 (−0.002–0.140)	0	0.42	0.46
Karagol	0.112 (**0.047**–0.199)	0.053 (−0.019–0.146)	0.233 (**0.020**–0.445)	0.025 (−0.024–0.095)	0	0.48
Kiziltepe	0.034 (−0.003–0.081)	0.061 (−0.029–0.167)	0.059 (−0.027–0.139)	0.026 (−0.030–0.110)	0.118 (**0.042**–0.188)	0

The lower diagonal represents the pairwise Fst (Nei, 1987) and the upper diagonal represents the mean proportion of shared alleles between sampling localities. The 95% confidence intervals in brackets for Fst values (1,000 bootstraps, estimates were lower CI limit >0 highlighted in bold font). L: low altitude, M: middle altitude, and H: high altitude.

Table 4 presents the mean proportions of shared alleles and the pairwise Fst among populations. The shared alleles proportions ranged from 38% (Eregli and Kiziltepe) to 52% (Madenkoy and Darbogaz), while the Fst varied from 0.025 (Karagol and Madenkoy) to 0.25 (Karagol and Darbogaz). Notably, the Fst between Uluskisla and Madenkoy was negative (−0.025), likely due to the high proportion of missing data in the Madenkoy samples. Altitudinal genetic differences in Fst were 0.04 (low to middle), 0.06 (middle to high), and 0.11 (low to high). Notably, the confidence intervals for the Fst estimates were very wide, spanning zero in all cases except for four values: Darboğaz-Eregli = 0.056 (0.014–0.095), Karagol-Eregli = 0.112 (0.047–0.199), Karagol-Darbogaz = 0.233 (0.020–0.445), Kiziltepe-Karagol = 0.118 (0.042–0.188).

The MANOVA revealed the only significant variation between the sampling localities at the level of 11.3% (*p*-value = 0.01), but the variation among altitudes (6%, *p*-value = 0.49), and among samples within the same altitude (1%, *p*-value = 0.76) were non-significant. Finally, the two runs of STRUCTURE (with and without the prior) both indicated the best model at K = 2 with the Delta K values of 44.840244 and 121.992245, respectively. These results suggest that while our dataset could potentially represent an admixture of two genetically distinct clusters (see Supplementary Table S4; Supplementary Figure S5), this weak genetic structure does not reflect the altitudinal variation.

Our analysis of genetic relatedness at the individual level using *related* (Pew et al., 2015) revealed that the average observed within-group relatedness across all localities was significantly higher compared to the expected values (*p*-value = 0.01). At the level of locality, Eregli, Darbogaz, and Kiziltepe showed higher than expected relatedness, while Ulukisla, Madenkoy, and Karagol showed lower than expected relatedness (Supplementary Figure S6). The Mantel tests between the microbiome composition and the individual genetic relatedness in each sampling locality showed no significant correlation (*r* < 0.09, *p*-values >0.05).

### 3.8 Thyroxine hormone levels

**Figure 5:**
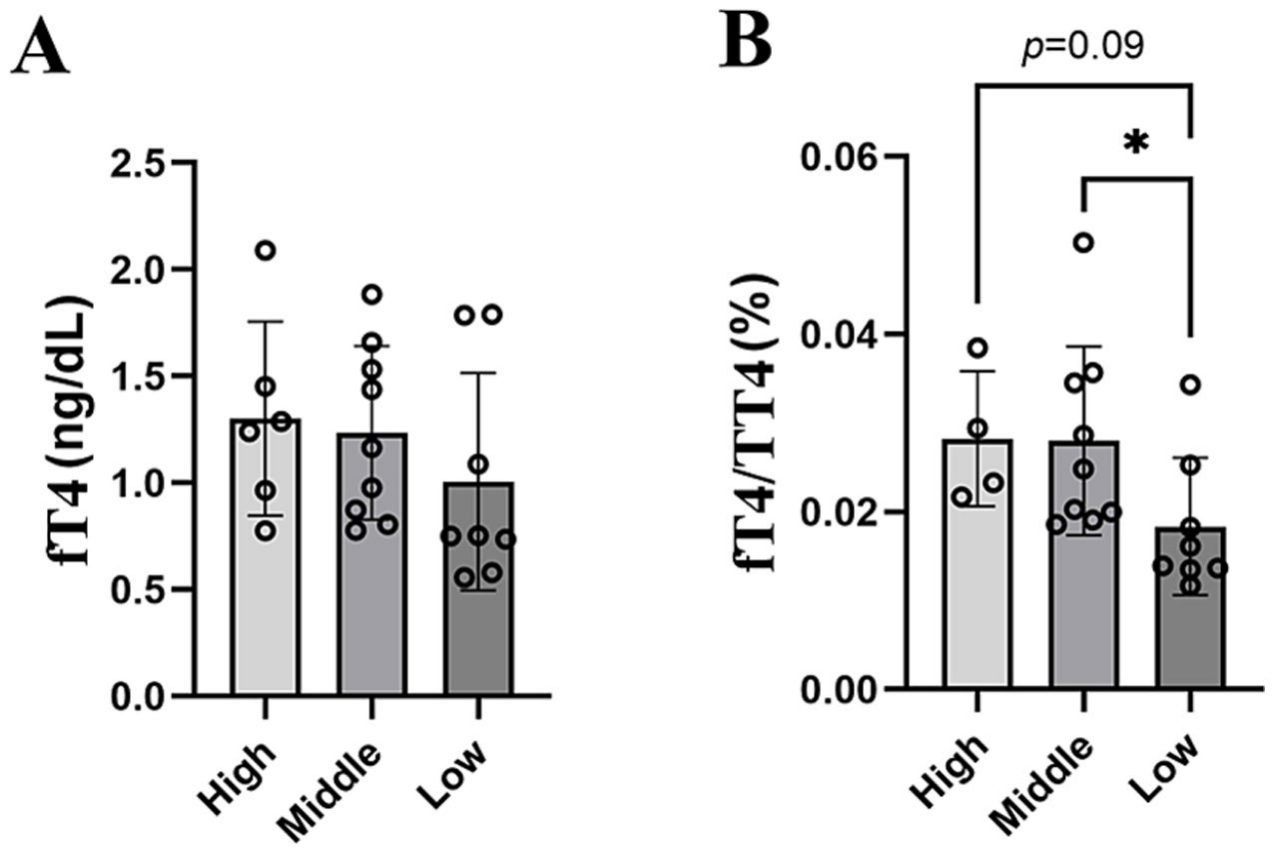
Thyroid hormone levels among altitudinal groups. (A) fT4 levels and (B) fT4/TT4 ratio.

To assess the effects of altitude on thyroid hormone levels and to account for additional factors known to influence thyroid hormone concentrations, we constructed multiple linear regression models with altitude, sex, and the body mass as explanatory variables. Altitude as a main effect exerted a significant effect on fT4 concentrations (*F* = 6.38, p-value = 0.021), while the sex had no effect (*F* = 0.0001, *p*-value = 0.98) and the body mass only had a marginal effect (*F* = 4.06, p-value = 0.059) on fT4. In contrast, none of the given factors had a significant effect on fT3. The main effects did not affect TT4 and TT3 concentrations, but the interaction of sex and altitude exerted a statistically significant effect on total hormone concentrations (Supplementary Table S5). Furthermore, the fT4/TT4 ratio was significantly lower in animals living at low altitudes compared to middle altitudes and showed a trend toward a significantly lower ratio compared to high altitudes (*p*-value = 0.09, Figure 5).

ANOVA and GLMMs revealed that there is a marginally significant effect of TT4 hormone levels on the Shannon and Simpson alpha diversity metrics. Specifically, TT4 levels showed negative correlation with alpha diversity metrics. The PERMANOVA performed on the Bray-Curtis distance matrix and GLMMs applied to the first axis of PCoA revealed that there is a significant effect of the FT4/TT4 levels on the microbiome beta diversity. However, while the correlation of the Bray-Curtis distance matrix correlated with the FT4/TT4 ratio positively, the PCoA axis showed negative correlation (Table 6; see Supplementary Table S6). Finally, the *DA.test* results revealed no significant correlation with the relative abundance of bacterial taxa and the levels of thyroid hormones.

**Table 6:** Summary of the tests conducted on the hormone levels and microbiome data.

	Measure	Test	Random effect	TT4 (ng/mL)	TT4/TT3	FT4/TT4
Alpha diversity	Shannon	ANOVA	No	0.055	0.064	0.038
	Simpson	ANOVA	No	0.026	0.071	0.023
	# of ASVs	GLMM	Locality	0.092	NS	NS
	Shannon	GLMM	Locality	0.021	0.057	0.062
	Simpson	GLMM	Locality	0.014	0.073	0.035
Beta diversity	Bray Dist	PERMANOVA	No	NS	NS	0.008
	PCoA Axis 1	GLMM	Locality	NS	NS	0.035

Only the significant or marginally insignificant tests are reported here, for all tests see Supplementary Table S6.

## 4 Discussion

Our study is the first to address the multiple aspects of altitude adaptation in the subterranean Blind Mole Rat (*Nannospalax xanthodon*) - a biomedically and evolutionarily important model organism. We found that altitude is indeed a key factor shaping the variation observed in (i) microbiome, (ii) diet, and (iii) thyroid hormone levels, whereas (iv) its population genetic structure reflects a fine-scale geographic pattern rather than the elevation gradient. We could not determine statistically whether the biological aspects (i-iv) also interact in a meaningful way *among* themselves, or if (i-iii) simply co-vary with the altitude. Below, we take advantage of the existing knowledge of the complex nature of the microbiome functioning and mammalian adaptation to high altitudes, in order to hypothesize on the possible mechanisms behind our findings.

### 4.1 Changes in bacterial taxa and phenotypes among the altitudinal groups

The ratio of Firmicutes/Bacteroidota (F/B) is linked to various host health, disease traits, and also linked with altitude according to most studies (Indiani et al., 2018; Jasirwan et al., 2021; Koliada et al., 2017; Mariat et al., 2009; An et al., 2023). A study on *Rhesus macaques* indicated that animals inhabiting high altitudes exhibit a higher abundance of Bacteroidota and a lower abundance of Firmicutes compared to those at lower altitudes (Wu et al., 2020). Another study examining the composition of the oral microbiome in Tibetans living at high (2800–3,650 m) and ultra-high (3650–4,500 m) altitudes reported a low F/B ratio in the populations at the highest altitude (Liu et al., 2021). This observation was speculated to be an adaptation to maintain normal blood pressure in hosts, considering the decrease in ambient oxygen and maximal oxygen consumption (VO2max) of the human body with altitude (Squires and Buskirk, 1982), and lower F/B ratio has been associated with a lower VO2max in another study (Durk et al., 2019). We observed the same pattern in AMBR (see Table 2; Figure 2A), which could suggest a role of the low F/B ratio in blood pressure maintenance. At the same time, the extreme specialization of the AMBR to subterranean lifestyle may create a very different context compared to the above listed examples. The ABMR responds to the hypoxia caused by low levels of O2 inside the tunnels rather than to its ambient atmospheric concentration, with the additional factors such as tunnel depth, soil type and permeability, animal own activity, etc. (see the *respiratory stress hypothesis* in (Arieli, 1979; Darden, 1972)) playing a role. Importantly, some studies have presented conflicting results regarding the link between the F/B ratio and altitude (ie., Li and Zhao, 2015; Zeng et al., 2017).

An increase in the abundance of strictly anaerobic bacteria at higher altitudes is a well-known phenomenon, for example in wild house mice (Suzuki et al., 2018) and humans (Lan et al., 2017), although such effects were mostly observed at much greater elevations (i.e., up to 4,500 m a.s.l.) compared to our study (the highest location was at 2900 m a.s.l.). The previously mentioned factors specific to the subterranean environment and AMBR lifestyle may also explain the lack of significant difference in the abundance of strictly anaerobic bacteria among our altitudinal groups. Even more striking is the complete absence from our 16S rRNA dataset of the anaerobic bacterial genus *Prevotella*, which was previously found to be strongly associated with increasing altitude in wild house mice (Suzuki et al., 2019), wild pikas (Li et al., 2016b), high altitude yaks, in Tibetan sheep (Zhang et al., 2016), and in humans (Li et al., 2016d; Lan et al., 2017), but see (Li and Zhao, 2015). The *Prevotella* is also present at ~3.5% in subterranean plateau zokors - another member of the family Spalacidae living at high altitudes (Hu et al., 2023). Our results as well as the previous studies suggest that the absence of *Prevotella* in Blind Mole Rats GM may actually be a common feature of the wild populations of these rodents: i.e. it was detected neither in the wild-caught *N. xanthodon* [(Kuang et al., 2022; Solak et al., 2023), nor in *N. ehrenbergi* (Kuang et al., 2022; Solak et al., 2023)], but Sibai et al. (2020) reported the first appearance of *Prevotella* in the fecal microbiomes on the AMBR only after they spent a month in captivity. Therefore, the importance of this bacterial taxon in the adaptation to high altitude does not seem to apply to our research system.

We showed that unlike the strictly anaerobic bacteria, the facultatively anaerobic ones were still significantly more abundant at high altitudes (Figure 3, Supplementary Figure S4). This functional group has a unique ability to grow in the presence or in the absence of oxygen and is thus well-adapted to changing environments (André et al., 2021). It is possible that extreme fluctuation of temperature and precipitation regime (including dense snow cover in winter) at high altitude also translates into a wide range of the ambient O2 concentrations experienced by the AMBR, and thus favors the facultatively anaerobic microbial elements.

The Ruminococcaceae bacterial family was found to be more prevalent during periods of limited energy availability in black howler monkeys, potentially compensating for reduced energy intake (Amato et al., 2015). This finding was further supported by (Wu et al., 2020), who reported an increased abundance of Ruminococcaceae in rhesus macaques inhabiting cold, high-altitude environments, suggesting a role in energy-saving. Interstingly, our results revealed not an increase but a decline in the relative abundance of Ruminococcaceae with increasing altitude (Table 3; Figure 2). This result could be attributed to the specifics of biology of the AMBR, in particular to its reduced tolerance of the heat stress (Šumbera et al., 2023). During the summer season when all our samples were collected, the animals at the low altitudes have to deal with much hotter and drier environment compared to the milder and wetter conditions at the higher elevation. This would likely cause a greater cumulative stress, causing the animals to reduce their activity and so exploit any available opportunity for greater energy efficiency, including the adjustment in the microbiome.

Muribaculaceae was reported to be linked with immune function (Sha et al., 2022; Huang et al., 2022) and to be more abundant in hypobaric hypoxic rats (Ma et al., 2023). A study on the plateau pikas found that Muribaculaceae is more abundant in the warm season and at high altitudes (Tang et al., 2023). Similarly, we observed a significant increase in the abundance of Muribaculaceae with increasing altitude, while all our samples were collected during the warmest time of the year. Previously, we showed that the high-altitude ABMRs from the same study area possess a greater innate immune response (Solak et al., 2020). We hypothesize that the higher abundance of Muribaculaceae could have contributed to a stronger immune function in high-altitude ABMRs. We note that apart from Muribaculaceae, the Akkermansiaceae (any many other bacteria) family has also been linked with immunoregulation and we only found it at low altitudes (Portincasa et al., 2024; Cani et al., 2022; Grenda et al., 2022). However, different bacterial species can stimulate different immune responses by activating different immune cells and pathways (Acosta and Alonzo, 2023; Zheng et al., 2020).

### 4.2 The effect of altitude on microbiome diversity and composition

In the context of laboratory rodent studies simulating high-altitude conditions, the relationship between GM diversity and altitude has yielded inconsistent results. A study on rats by (Tian et al., 2018) reported significantly higher GM alpha diversity at high altitudes, whereas (Zhang et al., 2018) observed slightly lower diversity (albeit not statistically significant). Higher GM alpha diversity at high altitudes was found in wild pikas (Li et al., 2019) and rhesus macaques (Wu et al., 2020; Zhao et al., 2018, 2023). Another study on wild mice ranging from sea level to 4,000 meters asl. Found a slight increase in diversity at high altitudes, although not statistically significant (Suzuki et al., 2018). It should be noted that none of these studies (except Suzuki et al., 2018) were conducted in continuous transects, i.e., the effect of latitude over altitude is unknown. The effect of altitude on the microbiome in the wild animal system thus remains understudied. Consequently, each study was only able to draw conclusions based on the specific biology of the host species and the characteristics of the study system.

In contrast to the above examples, our study design aimed at reducing the possible effect of the local geography and seasonal variation in the AMBR system within its natural habitat. We detected a very strong but non-linear effect of the altitude: the highest alpha-diversity was observed in both populations from the middle altitude group, while the low and high altitudes did not differ significantly (Figure 2C). The reason behind this is that the middle altitude in fact combines all the specific (i.e., significantly over-abundant) ASVs from both low and high altitudes (Table 4). A straightforward interpretation for such a pattern would be the existence of two polarized assemblies of the microbial taxa, at low and high elevations respectively, with the middle altitude serving as a bridge between them. Recall that the obligate subterranean lifestyle of the AMBR implies very slow and gradual dispersal (Rado et al., 1992). If we then assume that the transfer of the GM elements co-occurs with the host, the direct exchange between the low and the high altitudes would seem difficult: i.e. there is no direct pathway for the lowland bacteria to reach highland habitats (and vice versa) without first passing through the middle altitude. In addition, the middle altitude belt is expected to have relatively mild environmental conditions (i.e., less extreme fluctuation in temperature and humidity), which could also favor the higher microbiome diversity at the individual level. Verification of this intriguing hypothesis will require collecting additional data, accounting for multiple potential ecological factors that could act to ‘polarize’ the high and low GM communities.

The altitude is known to exert significant effect on microbial composition at the population level (beta diversity), as reported in previous studies on rodents (Li et al., 2019; Li et al., 2016a; Li et al., 2016b; Suzuki et al., 2018) and humans (Zeng et al., 2020; Lan et al., 2017; Li and Zhao, 2015). Most studies explained this effect as an indirect result of other altitude-related processes, such as variation in the host diet, and in host genotypes. In most cases it was difficult to eliminate the effect of geography / spatial variation while testing for the effect of the altitude. In our study, we attempted to minimize the effect of geography by collecting samples within a small area featuring a steep elevation gradient. A very strong altitude effect on the GM composition revealed was mostly caused by a single high-altitude locality (Kiziltepe) clustering far from the rest of the data (i.e., PCoA plot on Figure 2D). Further analysis using DESeq2 revealed that this particular population harbored the highest number (*n* = 15) of significantly overrepresented ASVs - at least three times that of the second high-altitude locality Karagöl (*n* = 4), which is only a few hundred meters lower in terms of absolute elevation (Table 1). The population residing at Kiziltepe had the lowest genetic diversity, but the host genetics is an unlikely factor to explain the microbiome difference there, given the overall lack of correlation between the GM and population genetic structure. Neither could we find any significant difference in diet composition between Kiziltepe and Karagöl, which undermines the role of diet on the microbiome composition, at least in the case of AMBR. In addition, we note that Kiziltepe is situated next to an open mining area active since 1825, primarily extracting minerals such as iron, lead, silver, and gold (Kahya, 2018; Anatolia, 2023). Previous studies have indicated a significant impact of heavy metals on the gut microbiota of mice (Breton et al., 2013). In the absence of other clues, we can suggest that the distinct composition of the gut microbiota observed in Kiziltepe could potentially be attributed to the influence of heavy metal exposure as well as the adaptation to high altitude.

### 4.3 The effect of diet and host genetics on microbiome composition

The relationship between microbiome and host diet is discussed in several studies (Scott et al., 2013; Conlon and Bird, 2014; Beam et al., 2021; Leeming et al., 2021) with majority of authors finding significant effect of diet in GM. Even though we found significant changes in microbiome (Figure 2) and diet composition (Figure 4) among the altitudes, we did not find any correlation between the GM at the level of ABMR sampling localities and their respective diet composition. Despite the fact that we found no significant link between the altitude and the plant taxonomic diversity in the ABMR diet, the composition of the latter was still affected by the altitude. This is generally expected, given a major effect of the altitude on vegetation. Seasonal, geographical or even cultural variation in the diet has been shown to have a strong influence on microbiome (Amato et al., 2015; Guo et al., 2021); (De Filippo et al., 2010), and we attempted to minimize such effects by performing sampling during the same season and within the same mountain range. Notably, the plant order Asparagales, which includes species with characteristic bulbous high-nutrient content roots, was only present in the AMBR diet at high-altitude localities (Figures 4A,B). The ABMRs are known to prefer the bulbous rhizomes and collect them for storage in their tunnels (Sözen, 2005). It is possible that the ABMRs, being food generalists, also possess a ‘generalist’ gut microbiota equally suitable to help with the digestion of different plant species.

A review on effect of host genetic control over microbiome reported that over 110 different genetic loci were found to be associated with the abundance of specific gut microbes (Bubier et al., 2021) and other studies found significant link between certain genes and the abundance of bacterial taxa in the gut (Org et al., 2015; Zhernakova et al., 2024). In addition, the microbiomes usually remain species-specific despite similar diets and shared habitat (Kaneko et al., 2023). We found no systematic effect of altitude on genetic differentiation, i.e., two distant populations at low altitude differed much more than any populations across the elevation gradient. This result may suggest a relatively recent colonization of the higher elevations in our study area. It was reported that within the same population, the family members or the ones with closer social network often share similar microbiota compared to unrelated individuals (Lee et al., 2011; Yatsunenko et al., 2012; Tims et al., 2013; Raulo et al., 2021). Another study found differences in specific microbial taxa between the social and solitary hyena species (Münger et al., 2018). Unlike in the highly social animals, the direct horizontal transmission of bacteria in the BMR is expected to be more difficult due to their solitary lifestyle. In line with this expectation, no correlation was observed between the host relatedness and microbiome in our data.

Several animal and human studies have found that sex significantly affects GM diversity and composition, though this pattern is not always consistent (Kim et al., 2020). We did not observe any significant differences in GM diversity and composition between female and male animals. This inconsistency extends to the diversity and composition of the diet. Previous studies have discussed the relative strength of sex’s impact compared to other factors, such as host genetic background, age, and diet, with sex generally found to have lower influence on shaping the GM (Kovacs et al., 2011; Elderman et al., 2018; Org et al., 2016). Our findings support the notion that the effect of sex on GM is not universal and environmental factors, host diet and genetics have a stronger effect on GM.

### 4.4 Thyroid hormone levels

Previously it has been reported that thyroid hormone levels in Middle East blind mole-rats are influenced by climatic conditions (Avivi et al., 2014). Other thyroid hormone parameters were found to be correlated with microbiome diversity in humans (Zheng et al., 2020; Virili et al., 2024). We found that the free thyroxine (fT4) levels increased significantly with altitude after correcting for sex and weight (Figure 5; Supplementary Table S5), while the fT4/TT4 ratio decreased in animals at low altitude. These findings suggest that in the animals living at lower elevations, a lower proportion of total T4 resources is recruited into the biologically-active free fraction. As thyroid hormones are positive regulators of thermogenesis, lower recruitment of T4 into the free form in animals living at lower altitudes might be linked to warmer ambient temperatures (Yau and Yen, 2020; Gerhardt et al., 2023), which reduces thermoregulatory constraints. Overall, these results point toward higher metabolic rate at high altitudes (Supplementary Table S5). As an indicator of metabolic rate, the fT4 levels might also explain the higher abundance of facultatively anaerobic bacteria at high altitudes (see above).

The microbiota can affect thyroid hormone levels by regulating iodine uptake, influencing the availability of essential micronutrients like selenium and zinc, and impacting the absorption of thyroid medications and overall thyroid function (Fröhlich and Wahl, 2019); (Knezevic et al., 2020). We did not find any correlation between the thyroid hormone levels and the relative abundance of bacterial taxa. At the same time, all of the alpha diversity metrics were found to correlate with TT4 levels (Table 6). Specifically, there was negative correlation with TT4 and a positive correlation with fT4/TT4, suggesting that the recruitment of T4 into its biologically-active form might contribute to an increase in the alpha diversity metrics. This is also in line with the negative correlation between TT4 levels and alpha diversity metrics, because circulating fT4 suppresses thyroid hormone synthesis and secretion via a negative feedback loop (Ortiga-Carvalho et al., 2016). Furthermore, PERMANOVA and GLMM on the first principle component of PCoA of Bray-Curtis similarities were found to be positively associated with fT4/TT4 (Table 6), similar to alpha diversity metrics. At this stage, it is not possible to establish whether there is a causal relationship between thyroid hormones and microbiome composition, or if both respond to the altitude in an independent manner. The relationship between metabolism and the GM is well-established (Martin et al., 2019), in particular between the metabolic rate and thyroid hormones (Mullur et al., 2014). In the case of ABMR, facilitating the nutrient breakdown could be necessary to fuel the thyroid hormone-driven thermogenesis - which in turn would put direct selective pressure on the GM landscape at higher altitudes.

## 5 Conclusion

This study explored the effect of altitude on the gut microbiome composition of ABMRs, across six localities and three altitudinal categories, considering the factors such as diet, thyroid hormone levels, and host genetics. Notable differences in the relative abundance of a number of bacterial taxa at different altitudes may reflect the specific roles these bacteria play in the complex adaptation of the host to the challenging mountain environment. The fact that the abundance of strictly anaerobic bacteria was unaffected by the altitude may reflect the relatively narrow range of absolute elevations in our study (1000–3,000 m a.s.l.), or it may be a specific feature of the host: the latter possibility is partly supported by the fact that the previously identified high altitude-linked genus *Prevotella* was absent from our data, in line with the previous studies done on the BMR. At the same time, the facultatively anaerobic bacteria were more prevalent in the high-altitude host specimens. We showed that the microbiome alpha-diversity reached its peak at the middle altitude, and that it incorporated elements from both lower and higher elevations. The beta-diversity correlated positively with the altitude. The altitude also affected the host diet composition, but not its alpha-diversity. These observations are unlikely to be caused by the host genetics, since we detected no clear association between the population genetic structure and the altitude, nor there was any correlation between the host relatedness and the microbiome composition nor diet. Thyroid hormone levels, specifically free thyroxine (FT4), increased almost linearly with altitude; however, no specific associations were found between bacterial ASVs and hormone levels. At the same time, the total thyroxine (TT4) levels did show a positive correlation with microbiome diversity. While some correlations between thyroid hormone components and microbiome beta diversity were identified, the nature of these relationships remains unclear.

### 5.1 Perspectives

Several properties of our study design could have unavoidably acted as caveats of the results presented. In particular, we tried to reduce the effect of seasonal variation on microbiome by sampling in late June to mid-July during both years when the fieldwork was performed. At this time of year, the high altitudes experience humid and colder conditions, but also feature a lusher vegetation compared to the low altitudes, which are both drier and hotter. The effect of season on the microbiome composition and diversity is well-known (Davenport et al., 2014; Hu et al., 2018; Jiang et al., 2021; Fan et al., 2022). While the results presented here offer a comprehensive snapshot of the microbiome, diet and thyroid hormones levels variation during the summer months, we cannot exclude a very different pattern if the samples were collected during other seasons. For example, the conditions at the lower elevation in the Taurus mountains during early spring resemble those found in mid-summer at high altitudes, which may profoundly influence the AMBR activity pattern as well as diet - which in turn may cause a major shift in the data collected across the entire elevation gradient. In the future, it would be preferable to have samples from other seasons in order to gain better insight into the microbiome and physiological dynamics in this system.

Rather than exclusively concentrating on microbial diversity and composition, future studies could also explore the functional profiling of bacterial ASVs specific to each altitude category using high-resolution metabarcoding data (i.e., full 16S rRNA sequencing), or full microbial genomes. Field experiments to measure the *in situ* metabolic rate of the animals might also help to reveal the interplay between microbiome and host physiology. Additionally, considering the distinct microbiome composition we found at Kiziltepe, further investigation could involve comparing the soil microbiome with the host microbiome to understand potential correlations. For dietary composition analysis, more specific genetic markers that focus on plants (e.g., rbcL, trnL, ITS) could offer higher taxonomic assignment resolution. High-resolution genomic data (e.g., ddRAD-seq or WGS) might unveil more subtle genetic divergence and relatedness patterms among the sampling localities and altitudes. Lastly, it would be extremely interesting to check if the patterns we found in AMBR will also be observed in other terrestrial animal species residing at different elevations in the Taurus mountains of Anatolia.

## Data Availability

16S, 18S rRNA phyloseq databases, and thyroid hormone datasets are available at FigShare (DOI: 10.6084/m9.figshare.25982311), and the microsatellite dataset is available in the Supplementary Tables. The 16S and 18S rRNA raw reads available at the European Nucleotide Archive under the project number of the study PRJEB76873 and the accession numbers for the samples are ERS20304461 - ERS20305352. The R code used to perform the analyses is available at GitHub (https://github.com/mrsolak/nannospalax_altitude).

## Footnotes

^1^Available at: https://drive5.com/uchime/gold.fa

^2^ https://www.r-project.org/
