# Supplementary material for: Altitude shapes gut microbiome composition accounting for diet, thyroid hormone levels, and host genetics in a subterranean blind mole rat
Source: Front Microbiol. 2024 Nov 1;15:1476845. doi: 10.3389/fmicb.2024.1476845 (PMC11565052; doi:10.3389/fmicb.2024.1476845)
Supplement: Supplementary file 1 [file Table_1.docx]

**Supplementary Tables:**

**Table S1.** List of the sequencing barcodes for each sample. Forward primer (F_Trus4) TGTACCTACGGGNGGCWGCAG and reverse primer GACTACHVGGGTATCTAATCC (R_Trus).

| sample_ID | altitude | locality | F_barcode | R_barcode |
| --- | --- | --- | --- | --- |
| E9-1 | low | eregli | TAGGCATG | CTCCTTAC |
| E9-1 | low | eregli | ATGCGCAG | AGCTAGAA |
| ER-1 | low | eregli | GTAGAGGA | AGAGGATA |
| ER-1 | low | eregli | CGATCAGT | CTAGTCGA |
| ER-2 | low | eregli | GCTCATGA | AGAGGATA |
| ER-2 | low | eregli | TGCAGCTA | CTAGTCGA |
| ER-3 | low | eregli | ATCTCAGG | AGAGGATA |
| ER-3 | low | eregli | CGATCAGT | ACTCTAGG |
| ER-4 | low | eregli | TAAGGCGA | CTCCTTAC |
| ER-4 | low | eregli | TGCAGCTA | ACTCTAGG |
| U01 | low | ulukisla | ATCTCAGG | CTCCTTAC |
| U01 | low | ulukisla | TCGACGTC | AGCTAGAA |
| U02 | low | ulukisla | TAAGGCGA | TATGCAGT |
| U02 | low | ulukisla | ACTCGCTA | ACTCTAGG |
| U03 | low | ulukisla | CGTACTAG | TATGCAGT |
| U03 | low | ulukisla | GGAGCTAC | ACTCTAGG |
| U04 | low | ulukisla | AGGCAGAA | TATGCAGT |
| U04 | low | ulukisla | GCGTAGTA | ACTCTAGG |
| U06 | low | ulukisla | TAGGCATG | AGAGGATA |
| U06 | low | ulukisla | ATGCGCAG | CTAGTCGA |
| U07 | low | ulukisla | CTCTCTAC | AGAGGATA |
| U07 | low | ulukisla | TAGCGCTC | CTAGTCGA |
| U08 | low | ulukisla | CGAGGCTG | AGAGGATA |
| U08 | low | ulukisla | ACTGAGCG | CTAGTCGA |
| U09 | low | ulukisla | AAGAGGCA | AGAGGATA |
| U09 | low | ulukisla | CCTAAGAC | CTAGTCGA |
| A03 | middle | madenkoy | CTCTCTAC | CTCCTTAC |
| A03 | middle | madenkoy | TAGCGCTC | AGCTAGAA |
| A04 | middle | madenkoy | CGAGGCTG | CTCCTTAC |
| A04 | middle | madenkoy | ACTGAGCG | AGCTAGAA |
| A05 | middle | madenkoy | AAGAGGCA | CTCCTTAC |
| A05 | middle | madenkoy | CCTAAGAC | AGCTAGAA |
| A07 | middle | madenkoy | GTAGAGGA | CTCCTTAC |
| A07 | middle | madenkoy | CGATCAGT | AGCTAGAA |
| A10 | middle | madenkoy | GCTCATGA | CTCCTTAC |
| A10 | middle | madenkoy | TGCAGCTA | AGCTAGAA |
| DB-1 | middle | darbogaz | GTAGAGGA | ATAGAGAG |
| DB-1 | middle | darbogaz | CGATCAGT | CGGAGAGA |
| DB-2 | middle | darbogaz | GCTCATGA | ATAGAGAG |
| DB-2 | middle | darbogaz | TGCAGCTA | CGGAGAGA |
| DB-3 | middle | darbogaz | ATCTCAGG | ATAGAGAG |
| DB-3 | middle | darbogaz | ACTGAGCG | ACTCTAGG |
| DB-4 | middle | darbogaz | TAAGGCGA | AGAGGATA |
| DB-4 | middle | darbogaz | ACTCGCTA | CTAGTCGA |
| DB-5 | middle | darbogaz | CGTACTAG | AGAGGATA |
| DB-5 | middle | darbogaz | CCTAAGAC | ACTCTAGG |
| DB-6 | middle | darbogaz | AGGCAGAA | AGAGGATA |
| DB-6 | middle | darbogaz | GCGTAGTA | CTAGTCGA |
| DB-7 | middle | darbogaz | TCCTGAGC | AGAGGATA |
| DB-7 | middle | darbogaz | CGGAGCCT | CTAGTCGA |
| DB-8 | middle | darbogaz | GGACTCCT | AGAGGATA |
| DB-8 | middle | darbogaz | TACGCTGC | CTAGTCGA |
| KZ-1 | high | kiziltepe | TAAGGCGA | ATAGAGAG |
| KZ-1 | high | kiziltepe | CGGAGCCT | ACTCTAGG |
| KZ-2 | high | kiziltepe | CGTACTAG | ATAGAGAG |
| KZ-2 | high | kiziltepe | GGAGCTAC | CGGAGAGA |
| KZ-3 | high | kiziltepe | AGGCAGAA | ATAGAGAG |
| KZ-3 | high | kiziltepe | TACGCTGC | ACTCTAGG |
| KZ-4 | high | kiziltepe | TCCTGAGC | ATAGAGAG |
| KZ-4 | high | kiziltepe | CGGAGCCT | CGGAGAGA |
| KZ-5 | high | kiziltepe | GGACTCCT | ATAGAGAG |
| KZ-5 | high | kiziltepe | ATGCGCAG | ACTCTAGG |
| KZ-6 | high | kiziltepe | TAGGCATG | ATAGAGAG |
| KZ-6 | high | kiziltepe | ATGCGCAG | CGGAGAGA |
| KZ-7 | high | kiziltepe | CTCTCTAC | ATAGAGAG |
| KZ-7 | high | kiziltepe | TAGCGCTC | CGGAGAGA |
| KZ-8 | high | kiziltepe | CGAGGCTG | ATAGAGAG |
| KZ-8 | high | kiziltepe | ACTGAGCG | CGGAGAGA |
| KZ-9 | high | kiziltepe | AAGAGGCA | ATAGAGAG |
| KZ-9 | high | kiziltepe | TAGCGCTC | ACTCTAGG |
| K01 | high | kiziltepe | CGTACTAG | AGGCTTAG |
| K01 | high | kiziltepe | GGAGCTAC | ATAGCCTT |
| K02 | high | kiziltepe | CGTACTAG | TCTTACGC |
| K02 | high | kiziltepe | GGAGCTAC | TAAGGCTC |
| K03 | high | kiziltepe | CGTACTAG | ATTAGACG |
| K03 | high | kiziltepe | GGAGCTAC | TCGCATAA |
| K04 | high | kiziltepe | AGGCAGAA | ATAGAGAG |
| K04 | high | kiziltepe | GCGTAGTA | CGGAGAGA |
| K05 | high | kiziltepe | AGGCAGAA | AGAGGATA |
| K05 | high | kiziltepe | GCGTAGTA | CTAGTCGA |
| K06 | high | kiziltepe | AGGCAGAA | CTCCTTAC |
| K06 | high | kiziltepe | GCGTAGTA | AGCTAGAA |
| K08 | high | kiziltepe | AGGCAGAA | TATGCAGT |
| K08 | high | kiziltepe | GCGTAGTA | ACTCTAGG |
| K09 | high | kiziltepe | AGGCAGAA | TACTCCTT |
| K09 | high | kiziltepe | GCGTAGTA | CTTAATAG |
| K10 | high | kiziltepe | AGGCAGAA | AGGCTTAG |
| K10 | high | kiziltepe | GCGTAGTA | ATAGCCTT |
| K11 | high | kiziltepe | AGGCAGAA | TCTTACGC |
| K11 | high | kiziltepe | GCGTAGTA | TAAGGCTC |

**Table S2.**  List of the sequencing barcodes for each sample. Forward primer 5'–GATYTGTCTGGTTVATTCCG–3' and reverse primer 5'–CATCACAGACCTGTTATYGC–3'.

| sample_ID | altitude | locality | F_barcode | R_barcode |
| --- | --- | --- | --- | --- |
| E9-1 | low | eregli | AAGAGGCA | CGGAGAGA |
| E9-3 | low | eregli | GCTCATGA | CGGAGAGA |
| E9-4 | low | eregli | ATCTCAGG | CGGAGAGA |
| ER-1 | low | eregli | CTCTCTAC | AGCTAGAA |
| ER-2 | low | eregli | CGAGGCTG | AGCTAGAA |
| ER-3 | low | eregli | AAGAGGCA | AGCTAGAA |
| ER-4 | low | eregli | GTAGAGGA | AGCTAGAA |
| U01 | low | ulukisla | TAGGCATG | CTAGTCGA |
| U03 | low | ulukisla | AGGCAGAA | CTAGTCGA |
| U06 | low | ulukisla | GGACTCCT | AGCTAGAA |
| U09 | low | ulukisla | TAGGCATG | AGCTAGAA |
| A03 | middle | madenkoy | TCCTGAGC | CTAGTCGA |
| A04 | middle | madenkoy | CGTACTAG | CGGAGAGA |
| A05 | middle | madenkoy | AGGCAGAA | CGGAGAGA |
| A06 | middle | madenkoy | TCCTGAGC | CGGAGAGA |
| A07 | middle | madenkoy | GGACTCCT | CGGAGAGA |
| A08 | middle | madenkoy | TAGGCATG | CGGAGAGA |
| A09 | middle | madenkoy | CTCTCTAC | CGGAGAGA |
| A10 | middle | madenkoy | GGACTCCT | CTAGTCGA |
| DB-1 | middle | darbogaz | TAAGGCGA | AGCTAGAA |
| DB-2 | middle | darbogaz | CGTACTAG | AGCTAGAA |
| DB-4 | middle | darbogaz | AGGCAGAA | AGCTAGAA |
| DB-8 | middle | darbogaz | TCCTGAGC | AGCTAGAA |
| K3 | high | karagol | TAAGGCGA | CTAGTCGA |
| K6 | high | karagol | CGTACTAG | CTAGTCGA |
| KZ-1 | high | kiziltepe | ATCTCAGG | AGCTAGAA |
| KZ-3 | high | kiziltepe | TAAGGCGA | ACTCTAGG |
| KZ-4 | high | kiziltepe | CGAGGCTG | CTAGTCGA |
| KZ-5 | high | kiziltepe | CGTACTAG | ACTCTAGG |
| KZ-6 | high | kiziltepe | GTAGAGGA | CTAGTCGA |
| KZ-7 | high | kiziltepe | GCTCATGA | CTAGTCGA |
| KZ-8 | high | kiziltepe | ATCTCAGG | CTAGTCGA |
| KZ-9 | high | kiziltepe | AGGCAGAA | ACTCTAGG |

**Table S3.** The microsatellite loci and the alleles. The first six loci are from Karanth et al. 2004 and the last seven loci are from Popa et al. 2014. Altitude category and sampling localities are abbreviated, see previous tables for exact names.

| ID | alt | loc | SpCA2.2 | SpCA2.8 | SpCA8.6 | SpCA1.1 | Sp4B1.1 | Sp4B1.2 | SL18 | SL51 | SL88 | SL96 | SL98 | SL53 | SL48 |
| --- | --- | --- | --- | --- | --- | --- | --- | --- | --- | --- | --- | --- | --- | --- | --- |
| E0 | low | ereg | 116140 | 130130 |  | 152152 | 160160 | 155155 | 194194 | 203205 |  | 106106 | 153155 | 261261 | 183183 |
| E1 | low | ereg | 140140 | 130130 | 234244 | 138152 | 152156 | 155159 |  | 203205 |  |  | 151151 |  |  |
| E2 | low | ereg | 114140 | 128130 | 244258 | 152152 | 148152 | 155155 |  |  |  |  |  |  |  |
| E9-1 | low | ereg | 140140 | 128128 | 228244 | 152152 | 160160 | 155159 | 198198 | 195209 |  | 106106 | 153155 | 253253 | 183183 |
| E9-2 | low | ereg | 114116 | 130130 | 228244 | 152152 | 156160 | 153159 | 198198 | 193209 | 133138 | 106106 | 153155 | 241241 | 183183 |
| E9-3 | low | ereg | 114116 | 128130 | 222222 | 152152 | 156160 | 153159 | 198198 | 195209 | 133138 | 106106 | 153155 | 241241 | 183183 |
| E9-4 | low | ereg | 114114 | 130130 | 222222 | 152152 | 156160 | 153159 | 198198 | 207207 | 138138 |  | 153155 |  | 183183 |
| E9-5 | low | ereg |  | 130130 | 228244 | 152164 |  | 153159 | 198198 | 195209 |  | 106106 | 155155 | 237263 |  |
| E9-6 | low | ereg | 114140 | 128130 | 222246 | 152164 | 156160 | 153159 | NA | 193193 |  |  |  |  |  |
| E9-7 | low | ereg | 140140 | 128130 | 222244 | 152152 | 156160 | 155159 | 198198 | 193193 |  | 106106 |  |  | 183183 |
| ER1 | low | ereg | 114140 | 128130 | 222258 | 138152 | 156156 | 153159 |  |  |  |  |  |  |  |
| ER2 | low | ereg | 118140 | 128130 | 246246 | 138152 | 156160 | 153159 |  |  |  |  |  |  |  |
| ER3 | low | ereg |  | 128130 | 246258 | 138152 |  | 153159 |  |  |  |  |  |  |  |
| ER4 | low | ereg | 114140 | 130130 | 222258 | 138152 | 156160 | 153159 |  |  |  |  |  |  |  |
| L10 | low | ereg | 114116 | 128130 | 222258 | 138152 | 156160 | 153159 | 192192 | 201201 |  | 104104 | 153155 | 241241 | 189189 |
| L2 | low | ereg |  | 128128 | 258258 | 138152 |  | 153153 | 192200 | 193195 | 133133 | 104104 | 151153 | 241241 |  |
| L6 | low | ereg | 114140 | 128130 | 234258 | 138152 | 156160 | 153153 | 192200 | 195207 | 133133 | 104104 | 153155 | 223241 | 189189 |
| L7 | low | ereg | 114140 | 128128 | 246258 | 138152 | 156156 | 153159 | 192200 |  |  | 104104 |  |  |  |
| U1 | low | ulu | 140142 | 128130 | 226248 | 152152 | 152156 | 155155 | 196198 | 209209 |  | 106106 | 155157 | 265265 | 183183 |
| U2 | low | ulu | 142142 | 128130 | 226248 | 152152 | 152156 | 155155 | 194198 | 193193 | 133138 | 106106 | 155157 | 237237 | 183183 |
| U3 | low | ulu | 140142 | 130130 | 222258 | 158158 | 156164 | 155159 | 194196 | 193209 | 133138 | 106106 | 153157 | 257265 | 183183 |
| U4 | low | ulu | 114140 | 130130 | 242248 | 164164 | 152164 | 155159 | 202202 | 209209 |  | 106106 | 151153 | 237237 | 183183 |
| U5 | low | ulu | 114140 | 128130 | 248248 | 152152 | 152160 | 155159 | 194198 | 193201 |  | 106106 | 153157 | 237237 | 171183 |
| DB1 | mid | dar | 114140 | 128128 | 246258 | 138152 | 156168 | 155159 |  |  |  |  |  |  |  |
| DB2 | mid | dar | 116140 | 128128 | 246258 | 138152 | 164164 | 155159 |  |  |  |  |  |  |  |
| DB3 | mid | dar | 116140 | 128128 | 246258 | 152152 | 156156 | 155159 |  |  |  |  |  |  |  |
| DB4 | mid | dar |  | 128128 | 246258 | 138152 |  | 155159 |  |  |  |  |  |  |  |
| DB5 | mid | dar | 114140 | 130130 | 246258 | 138152 | 156168 | 153159 |  |  |  |  |  |  |  |
| DB6 | mid | dar | 110140 | 128128 | 246258 | 152152 | 164168 | 155159 |  |  |  |  |  |  |  |
| DB7 | mid | dar | 110140 | 128130 | 246258 | 152156 | 156164 | 155159 |  |  |  |  |  |  |  |
| DB8 | mid | dar | 116140 | 130130 | 232246 | 158158 | 156168 | 153159 |  |  |  |  |  |  |  |
| A10 | mid | mad | 114140 | 128130 | 234234 | 138152 | 152152 | 155155 |  | 193195 |  |  | 155155 |  |  |
| A4 | mid | mad | 118140 | 128128 | 246258 | 138152 | 156164 | 155159 | 194198 | 203203 |  | 106106 |  |  |  |
| A5 | mid | mad | 118140 | 128130 | 248248 | 152152 | 152164 | 155159 | 194198 |  | 145145 | 106106 |  |  |  |
| A6 | mid | mad | 110140 | 130130 | 222258 | 138152 | 152164 | 155159 | 196200 | 195207 | 145145 | 106106 | 151151 | 263263 |  |
| A7 | mid | mad | 140142 | 130130 | 222258 | 138152 | 138152 | 155155 | 198200 | 203211 | 135135 | 106106 | 155155 | 241263 | 171183 |
| A8 | mid | mad | 140142 | 130130 | 242258 | 138152 | 152164 | 155159 | 192200 | 133133 | 104104 | 153153 | 223241 | 189189 |  |
| A9 | mid | mad |  | 130130 | 242246 | 138152 |  | 155159 | 200200 | 209209 | 133133 | 104104 | 153153 | 241241 |  |
| H1 | hi | kara |  | 130130 | 234258 | 138152 |  | 155159 | 200200 | 201203 | 161161 | 106106 | 151151 | 253253 | 183183 |
| H4 | hi | kara | 114140 | 128130 | 246258 | 138152 | 160164 | 155159 | 168168 | 205205 | 145169 | 153155 | 253253 | 183183 |  |
| H5 | hi | kara |  | 130130 | 222258 | 138152 |  | 155159 | 168186 | 205205 | 145147 | 122122 | 155155 |  | 183183 |
| H7 | hi | kara | 114140 | 130130 | 232234 | 138152 | 164164 | 155159 | 198198 | 209209 | 145147 | 122122 |  |  |  |
| K1 | hi | kara |  | 130130 | 232246 | 152152 |  | 155159 | 192198 | 193193 |  |  |  |  |  |
| K10 | hi | kara |  | 130130 | 234246 | 152152 |  | 155159 | 202202 | 201205 | 135145 | 106106 | 151151 | 261261 | 183183 |
| K11 | hi | kara | 140142 | 130130 | 246246 | 152158 | 164164 | 155159 | 194198 | 201205 | 135145 | 106106 | 155157 |  | 183183 |
| K12 | hi | kara | 140142 | 130130 | 226234 | 152158 | 152164 | 155159 | 200200 | 205205 | 138146 |  |  | 241263 | 171183 |
| K3 | hi | kara |  | 130130 | 234246 | 152152 |  |  | 192198 | 193207 | 106106 | 155155 | 257257 |  |  |
| K4 | hi | kara | 140140 | 130130 | 246246 | 152158 | 160160 | 155155 | 198198 | 193193 | 133133 | 106106 | 151155 | 241241 | 183183 |
| K5 | hi | kara | 140140 | 130130 | 232246 | 152158 | 160164 | 155159 | 192192 | 201207 |  | 106106 | 155155 | 257263 |  |
| K6 | hi | kara | 114140 | 130130 | 246246 | 152158 | 164164 | 155159 | 192192 | 207207 |  | 106106 | 155157 |  |  |
| K7 | hi | kara | 110140 | 130130 | 246246 | 152170 | 160160 | 155159 | 200200 | 203203 | 138145 | 106106 | 151157 | 263263 | 183183 |
| K8 | hi | kara | 140142 | 130130 | 234246 | 152170 | 160160 | 155159 | 194198 | 201201 | 135145 | 106106 | 155155 | 261261 |  |
| K9 | hi | kara |  | 130130 | 234246 | 152170 |  | 155159 | 194202 | 203205 | 138145 | 106106 | 151153 | 263263 | 183183 |
| KZ1 | hi | kiz |  | 130130 | 228228 | 158158 |  | 155155 |  |  |  |  |  |  |  |
| KZ2 | hi | kiz | 114140 | 128130 | 222258 | 158158 | 164164 | 155159 |  |  |  |  |  |  |  |
| KZ3 | hi | kiz | 140142 | 128128 | 258258 |  | 164164 | 155155 |  |  |  |  |  |  |  |
| KZ4 | hi | kiz |  | 130130 | 228246 |  |  |  |  |  |  |  |  |  |  |
| KZ5 | hi | kiz | 114140 | 130130 | 246258 | 146158 | 164164 | 155159 |  |  |  |  |  |  |  |
| KZ6 | hi | kiz | 114140 | 128130 | 258258 | 146152 | 164164 | 155159 |  |  |  |  |  |  |  |
| KZ7 | hi | kiz | 114140 | 130130 | 228248 | 152152 | 164164 | 155155 |  |  |  |  |  |  |  |
| KZ8 | hi | kiz | 114140 | 130130 | 246248 | 158158 | 164164 | 155155 |  |  |  |  |  |  |  |
| KZ9 | hi | kiz | 140140 | 128128 | 246258 | 152152 | 116164 | 155155 |  |  |  |  |  |  |  |
| M2 | hi | kiz | 114140 | 128130 | 246258 | 152152 | 164164 | 155155 |  | 207209 |  |  | 153155 |  | 183183 |
| M3 | hi | kiz | 114140 | 128130 | 248258 | 138152 | 164164 | 155155 | 198198 | 195209 | 131131 | 106106 | 155155 | 253253 | 183183 |
| M4 | hi | kiz | 114140 | 130130 | 228258 | 138152 | 164164 | 155155 | 192198 | 195195 |  | 106106 | 153155 | 253253 | 183183 |

**Table S4.** The Evanno table output from Structure Harvester software. A: without sampling locality as prior and B: with sampling locality as prior. Tables sorted for highest Delta K values.

| **A** | K | Reps | Mean LnP(K) | Stdev LnP(K) | Ln'(K) | \|Ln''(K)\| | Delta K |
| --- | --- | --- | --- | --- | --- | --- | --- |
|  | 2 | 5 | -1498.46 | 0.8081 | 110.68 | 98.58 | 121.992245 |
|  | 4 | 5 | -1482.1 | 34.2708 | 4.26 | 29.96 | 0.874213 |
|  | 3 | 5 | -1486.36 | 10.6409 | 12.1 | 7.84 | 0.736782 |
|  | 5 | 5 | -1447.88 | 49.7672 | 34.22 | 28.82 | 0.579096 |
|  | 1 | 5 | -1609.14 | 0.6107 | NA | NA | NA |
|  | 6 | 5 | -1442.48 | 66.5383 | 5.4 | NA | NA |
|  |  |  |  |  |  |  |  |
| **B** | K | Reps | Mean LnP(K) | Stdev LnP(K) | Ln'(K) | \|Ln''(K)\| | Delta K |
|  | 2 | 6 | -1500.1833 | 1.7566 | 109.25 | 78.766667 | 44.840244 |
|  | 5 | 6 | -1456.3833 | 21.9581 | 13.633333 | 242.733333 | 11.054393 |
|  | 3 | 6 | -1469.7 | 10.9771 | 30.483333 | 30.8 | 2.80585 |
|  | 4 | 6 | -1470.0167 | 14.8206 | -0.316667 | 13.95 | 0.941259 |
|  | 1 | 6 | -1609.4333 | 1.3186 | NA | NA | NA |
|  | 6 | 6 | -1685.4833 | 332.5457 | -229.1 | NA | NA |

**Table S5.** Summary of multiple linear regression models calculated to determine the best predictors for differences in thyroid hormone levels. The best fitting model was identified based on the Akaike information criterion (AIC) for each hormone (smaller value = preferred model).

|  | Model | Factors | *F* | *p* | **AIC** |
| --- | --- | --- | --- | --- | --- |
| fT4 | 1 | Altitude | 6.38 | 0 | **-71.8** |
|  |  | Sex | 0 | 1 |  |
|  |  | Weight | 4.06 | 0.1 |  |
|  | 2 | Altitude : Sex | 0.96 | 0.3 | **-69.7** |
|  |  | Altitude : Weight | 0.56 | 0.5 |  |
|  |  | Sex : Weight | 0.11 | 0.7 |  |
| fT3 | 1 | Altitude | 0.03 | 0.9 | **5.88** |
|  |  | Sex | 1.45 | 0.2 |  |
|  |  | Weight | 0 | 1 |  |
|  | 2 | Altitude : Sex | 8.19 | 0.4 | **5.74** |
|  |  | Altitude : Weight | 3.1 | 0.3 |  |
|  |  | Sex : Weight | 0 | 1.2 |  |
| TT4 | 1 | Altitude | 0.26 | 0.6 | **157** |
|  |  | Sex | 0.02 | 0.9 |  |
|  |  | Weight | 2.91 | 0.1 |  |
|  | 2 | Altitude : Sex | 4.9 | 0 | **155** |
|  |  | Altitude : Weight | 2.43 | 0.1 |  |
|  |  | Sex : Weight | 0.01 | 0.9 |  |
| TT3 | 1 | Altitude | 0.32 | 0.6 | **-49.3** |
|  |  | Sex | 0.28 | 0.6 |  |
|  |  | Weight | 0.44 | 0.5 |  |
|  | 2 | Altitude : Sex | 6.18 | 0 | **-55.5** |
|  |  | Altitude : Weight | 1.37 | 0.3 |  |
|  |  | Sex : Weight | 1.82 | 0.2 |  |

**Table S6.** All the tests conducted tests on hormone levels and microbial dataset. Green cells represent positive and orange cells represent negative correlation.

|  | Measure | Test | Random Effect | FT4 (ng/mL) | FT3 (ng/mL) | TT4 (ng/mL) | TT3 (ng/mL) | FT4/FT3 | TT4/TT3 | FT4/TT4 | FT3/TT3 |
| --- | --- | --- | --- | --- | --- | --- | --- | --- | --- | --- | --- |
| Abundance | Relative Abundance of Phyla | DA.kru | No | NS | NS | NS | NS | NS | NS | NS | NS |
| Alpha Diversity | # of ASVs | ANOVA | No | NS | NS | NS | NS | NS | NS | NS | NS |
|  | Shannon | ANOVA | No | NS | NS | 0.055 | NS | NS | 0.064 | 0.0379 | NS |
|  | Simpson | ANOVA | No | NS | NS | 0.0256 | NS | NS | 0.071 | 0.0233 | NS |
|  | # of ASVs | GLMM | Locality | NS | NS | 0.092 | NS | NS | NS | NS | NS |
|  | Shannon | GLMM | Locality | NS | NS | 0.0212 | NS | NS | 0.057 | 0.062 | NS |
|  | Simpson | GLMM | Locality | NS | NS | 0.0143 | NS | NS | 0.073 | 0.035 | NS |
| Beta Diversity | Bray Dist | PERMANOVA | No | NS | NS | NS | NS | NS | NS | 0.008 | NS |
|  | Bray Dist | MDMR | Locality | NS | NS | NS | NS | NS | NS | NS | NS |
|  | PCoA Axis 1 | GLMM | Locality | NS | NS | NS | NS | NS | NS | 0.035 | NS |
|  | PCoA Axis 2 | GLMM | Locality | NS | NS | NS | NS | NS | NS | NS | NS |
