# Supplementary material for: Altitude shapes gut microbiome composition accounting for diet, thyroid hormone levels, and host genetics in a subterranean blind mole rat
Source: Front Microbiol. 2024 Nov 1;15:1476845. doi: 10.3389/fmicb.2024.1476845 (PMC11565052; doi:10.3389/fmicb.2024.1476845)
Supplement: Supplementary file 2 [file Data_Sheet_1.docx]

**Supplementary Figures**


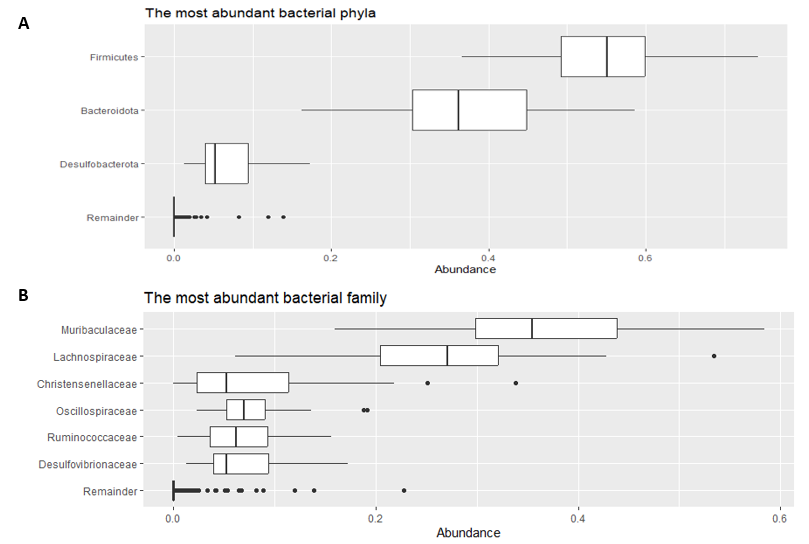


**Figure S1:** **A:** The most abundant bacterial phyla and **B:** The most abundant bacterial families (X–axis represents % of the abundance of all reads and “remainder” represents the taxa with <1) % abundance).


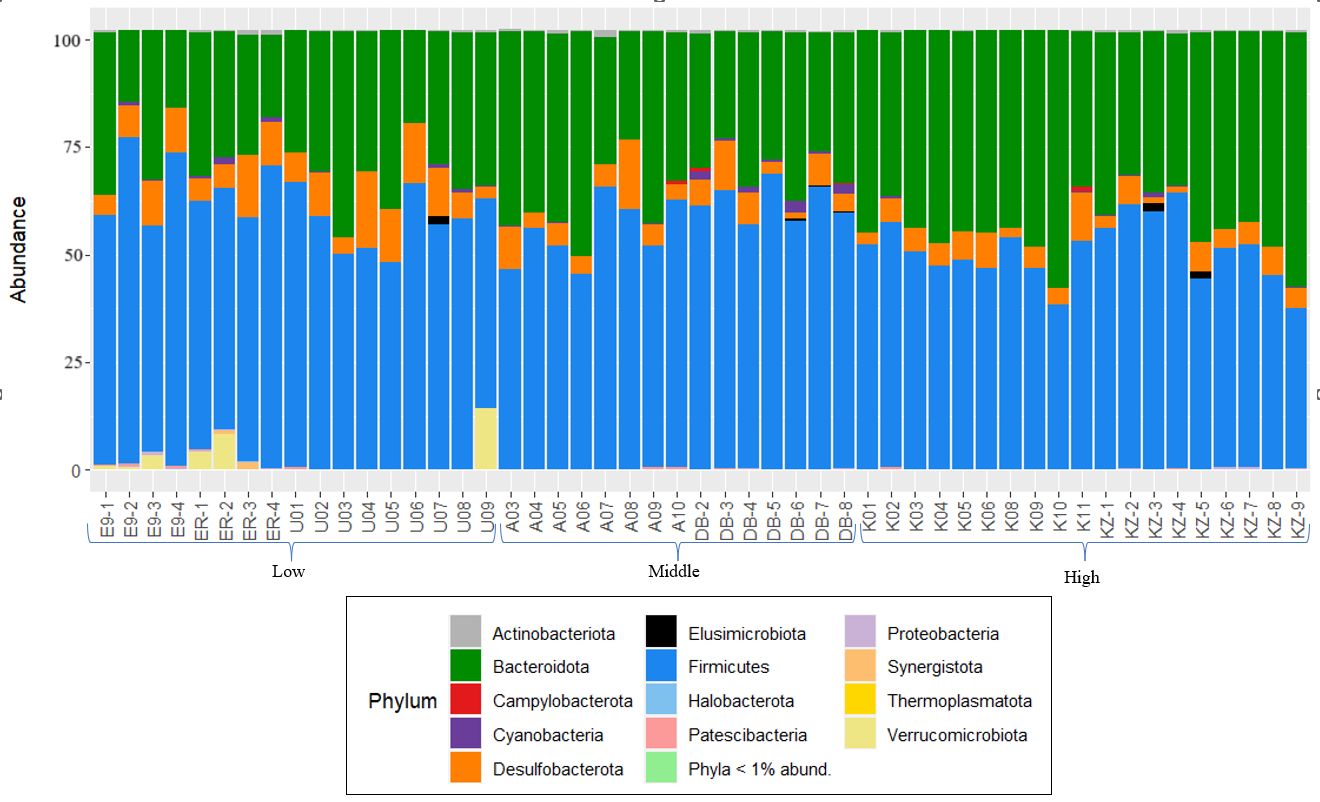


**Figure S2:** The relative abundance of bacterial phyla among individuals (Y–axis represents % of the abundance of all reads).


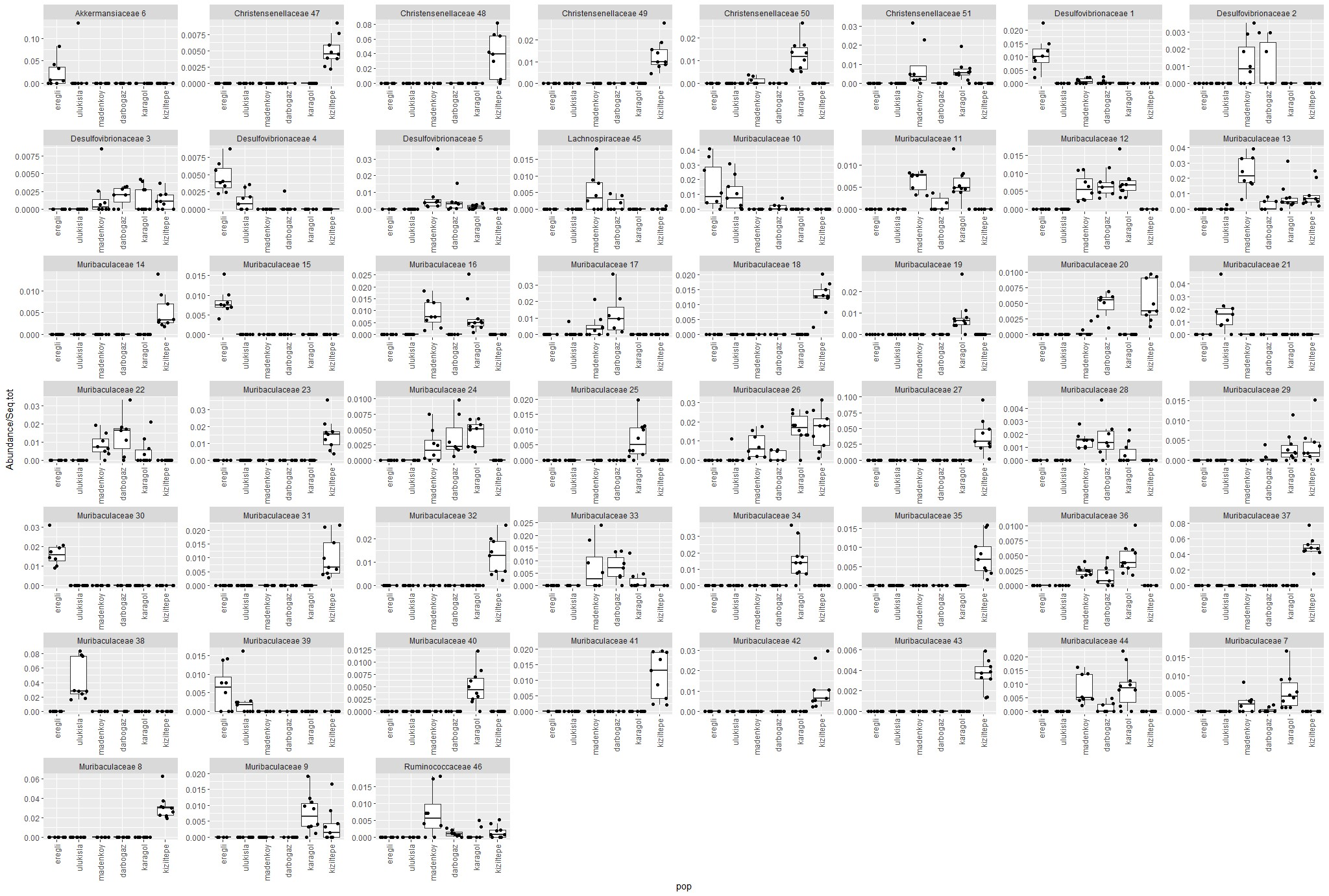


**Figure S3.** The plot shows the bacterial ASVs with significant relative abundance among sampling localities.


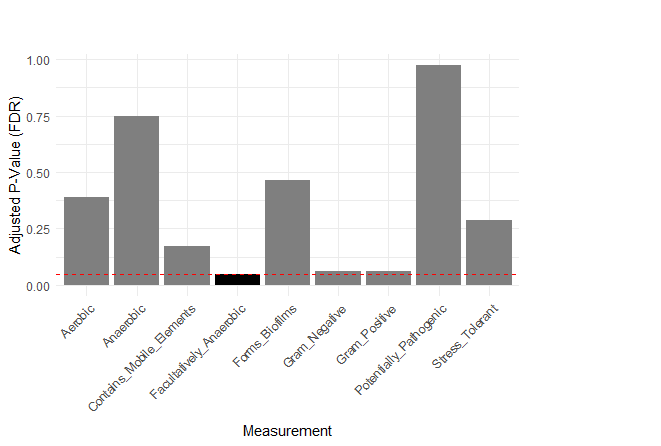


**Figure S4.** The barplot displays adjusted p-values (FDR-corrected) for each measurement, indicating the significance of differences in bacterial phenotypes between altitudinal groups. The dashed red line indicates the significance level which is 0.05. The black bar represents the significant p value.

**
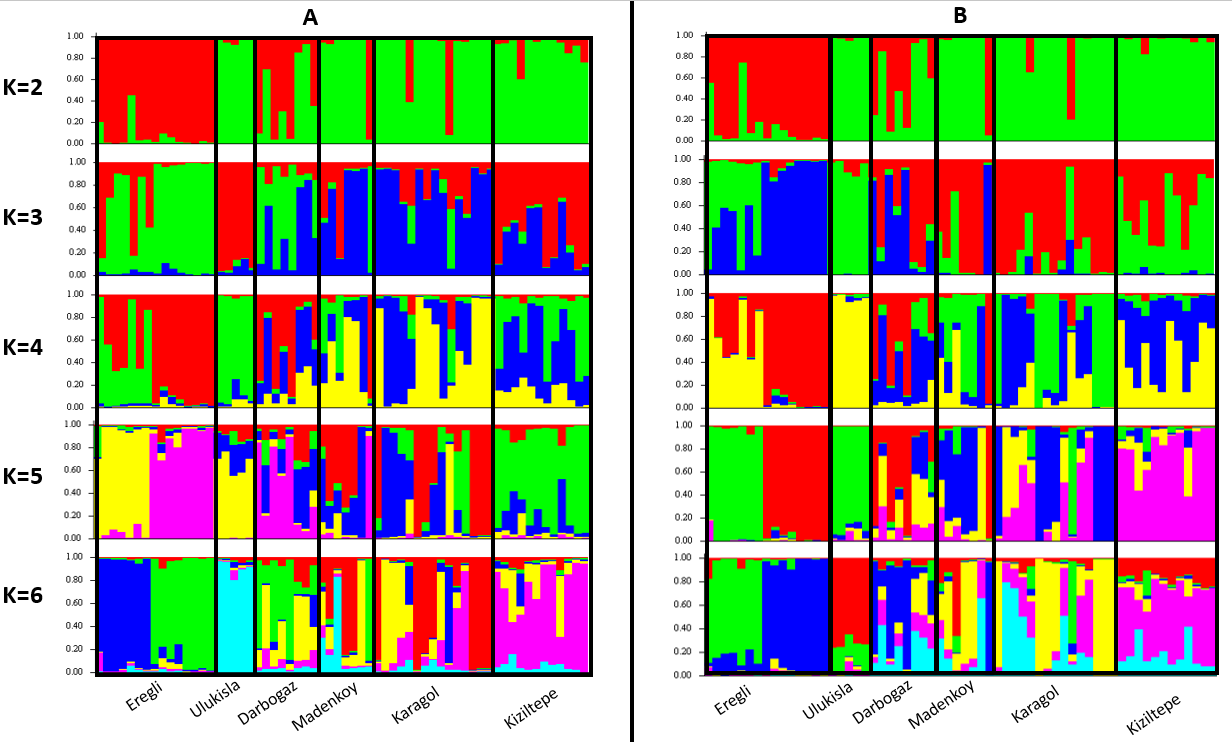
**

**Figure S5**. Barplots showing the results of STRUCTURE analyses. The first column contains the bar plots without sampling locality as prior and the second column contains the barplot with sampling locality as prior.

**
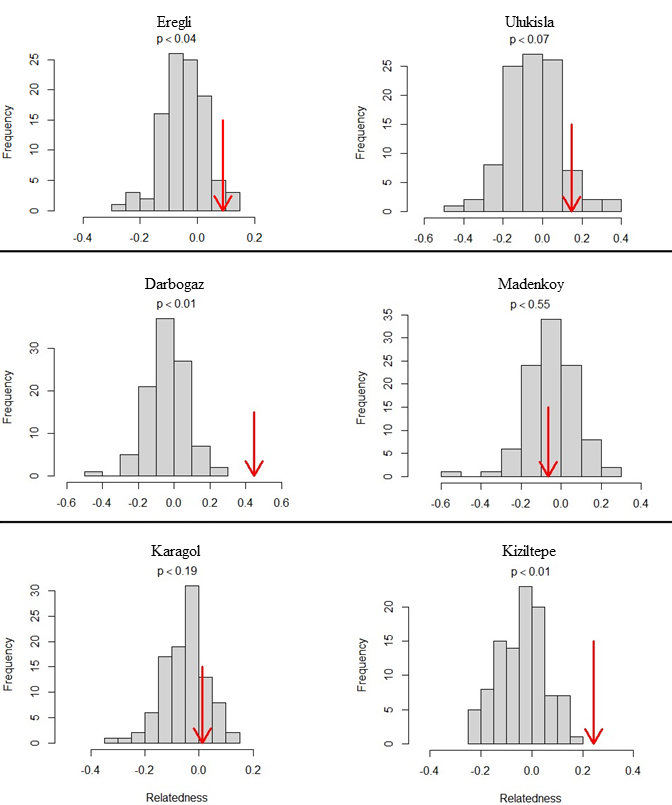
**

**Figure S6**. Comparison of expected and observed relatedness estimates (lynchli). Histogram represents the expected relatedness values and red arrow indicating where the observed value lies. P-values indicating expected values were greater than or equal to the observed value.
